# Beyond traditional assessments of cognitive status: Exploring the potential of spatial navigation tasks

**DOI:** 10.3758/s13428-026-02998-y

**Published:** 2026-04-17

**Authors:** Giorgio Colombo, Karolina Minta, Tyler Thrash, Jascha Grübel, Jan Wiener, Marios Avraamides, William R. Taylor, Christoph Hölscher, Victor R. Schinazi

**Affiliations:** 1 Future Health Technologies, Singapore-ETH Centre, Campus for Research Excellence and Technological Enterprise (CREATE), Singapore, 138602 Singapore; 2 Chair of Cognitive Science, ETH Zürich, 8092 Zurich, Switzerland; 3 Department of Biology, Saint Louis University, St. Louis, MO USA; 4 Department of Network and Data Science, Central European University, Vienna, Austria; 5 Department of Psychology, Bournemouth University, Bournemouth, UK; 6 Department of Psychology, University of Cyprus, Nicosia, Rise Nicosia Cyprus; 7 Institute for Biomechanics, Department of Health Sciences and Technology, ETH Zürich, Zurich, Switzerland; 8 Department of Psychology, Bond University, Robina, QLD 4226 Australia

**Keywords:** Spatial Navigation, Neuropsychology, Cognitive Status, Aging, Digital Cognitive Assessment, Psychometrics

## Abstract

Deficits in spatial and navigation abilities are among the earliest signs of dementia. Yet, traditional neuropsychological tests primarily target memory and attention. The Spatial Performance Assessment for Cognitive Evaluation (SPACE) is a novel gamified digital assessment for iPads that uses various spatial tasks to detect early deficits in spatial navigation abilities indicative of cognitive impairment. In this study, 348 participants aged 21–76 completed the Montreal Cognitive Assessment (MoCA), SPACE, and a sociodemographic and health questionnaire. We investigated whether SPACE could predict scores on the MoCA beyond known risk factors for cognitive impairment. Using a factor analysis, we then assessed whether SPACE could complement the MoCA by capturing latent variables independent of MoCA scores that represent additional spatial aspects of cognitive functioning. Results from a hierarchical regression revealed that the pointing and perspective-taking tasks in SPACE significantly predicted MoCA scores beyond age and gender. Surprisingly, none of the risk factors predicted MoCA scores. The factor analysis revealed that the MoCA and perspective-taking contributed to a separate factor from the other navigation tasks in SPACE. We also provide normative data on age and gender for each task in SPACE, which can serve as benchmarks for future studies to identify individuals at risk.

**Supplementary Information:**

The online version contains supplementary material available at 10.3758/s13428-026-02998-y.

## Introduction

Dementia affects 55 million people worldwide (World Health Organization, 2023), and this number is projected to increase to 152 million by 2050, posing a significant global threat to the healthcare system (Patterson, 2018). Alzheimer’s disease (AD) is the most common form of dementia and ranks as the fifth leading cause of death for those aged 65 and older (National Center for Health Statistics, 2021). The total economic burden of AD is projected to reach $3.3 trillion by 2060 (Nandi et al., 2024), including the costs of healthcare, long-term care, and informal caregiving (Alzheimer’s Association, 2024; Rajan et al., 2021). The early stage of dementia, known as mild cognitive impairment (MCI), often goes undiagnosed due to its mild symptoms and gradual onset. This stage presents a critical window for deploying sensitive assessments that may help reduce economic and societal costs via early intervention. While researchers have identified several risk factors associated with MCI and AD (Livingston et al., 2020), other predictors, such as spatial navigation performance, may complement existing assessments.

In addition to unmodifiable risk factors such as age (Alzheimer’s Disease Fact Sheet, 2023; Hebert et al., 2010), APOE genetic status (Kunz et al., 2015), and family history (Alzheimer’s Association, 2024; Hebert et al., 2013), the Lancet Commission has identified 14 modifiable risk factors (e.g., education, depression, physical inactivity) that account for nearly half of the dementia cases globally (Livingston et al., 2024). Previous research has found that these factors and other related risks are associated with the outcomes of widely used clinical assessments for cognitive status, including the Montreal Cognitive Assessment (MoCA; Bugallo-Carrera et al., 2024; Dale et al., 2018; Del Brutto et al., 2015; Dupuis et al., 2015; Freire et al., 2017; Jia et al., 2021; Nasreddine et al., 2005; Zawar et al., 2022) and the Mini-Mental State Examination (MMSE; Folstein, 1983; Heymann et al., 2016; Jia et al., 2021; Kim, 2022; Lv et al., 2024; Paterniti et al., 2002; Xu et al., 2009). Specifically, worse performance on clinical assessments is associated with older age (Bugallo-Carrera et al., 2024; Dale et al., 2018; Jia et al., 2021), being female (Bugallo-Carrera et al., 2024; Jia et al., 2021), but see (Dale et al., 2018), less education (Bugallo-Carrera et al., 2024; Dale et al., 2018), worse health status (Dale et al., 2018), physical inactivity (Iso-Markku et al., 2024), higher depression (Bugallo-Carrera et al., 2024; Byers & Yaffe, 2011; Dale et al., 2018; Del Brutto et al., 2015), more anxiety and stress (Del Brutto et al., 2015; Potvin et al., 2011), history of alcohol consumption (Heymann et al., 2016; Xu et al., 2009), smoking (Jia et al., 2021), sleep (McSorley et al., 2019; Zawar et al., 2022), and lower hearing or vision (Dupuis et al., 2015). The MoCA is often preferred to the MMSE because of its greater sensitivity in the detection of MCI and AD (Jia et al., 2021; Julayanont & Nasreddine, 2017; Pinto et al., 2019; Tsai et al., 2016) and, in clinical practice, provides a cheaper and less invasive alternative to a full neuropsychological assessment and the measurement of biomarkers of neurodegeneration.

Critically, biomarkers such as tau and β-amyloid (Aβ) are known to accumulate in brain regions that are essential for spatial navigation (Fyhn et al., 2004; Weisberg et al., 2014), especially the hippocampus and entorhinal cortex (Barthélemy et al., 2020; Chételat et al., 2010; Jack & Holtzman, 2013; Jack et al., 2010; Schmidt-Hieber & Häusser, 2013). Specifically, place cells in the hippocampus and grid cells in the entorhinal cortex have been found to be key components in encoding spatial locations and tracking positional changes during navigation (Epstein et al., 2017; Hafting et al., 2005; McNaughton et al., 2006; O’Keefe & Nadel, 1979). These findings suggest that spatial ability may be an important predictor of future cognitive status. Indeed, previous research has shown that spatial abilities are among the first skills to deteriorate as a consequence of AD (Castegnaro et al., 2023; Coughlan et al., 2018; deIpolyi et al., 2007; Hort et al., 2007; Howett et al., 2019; Segen et al., 2022). Although the MoCA and neuropsychological assessments emphasize working memory and executive functions and include some basic visuospatial tasks, a more comprehensive assessment of spatial and navigation abilities may further improve the sensitivity of these assessments. Notably, visuospatial tasks often focus on the relations among items at the micro-scale and therefore tap only on a subset of the skills required to navigate a large environment. Navigation in large environments requires additional skills such as the apprehension and integration of spatial information from multiple viewpoints and awareness of one’s own movement through space (Hegarty et al., 2006; Meneghetti et al., 2021). Here, researchers have used several spatial tasks to predict MCI and AD with varying degrees of success (Berron et al., 2024a; Chan et al., 2016; Coughlan et al., 2019; Hort et al., 2007; Rekers & Finke, 2024; Tu et al., 2015; van der Ham et al., 2020; Wiener et al., 2020) but have not systematically investigated the manner in which these spatial tasks can contribute to common cognitive assessments.

In the present study, we explore how spatial navigation performance, as measured by the Spatial Performance Assessment for Cognitive Evaluation (SPACE), relates to general cognitive functioning across the adult lifespan. SPACE is a novel gamified digital assessment that combines a variety of spatial tasks from previous research and is designed to identify deficits in spatial and navigation abilities indicative of early signs of cognitive impairment (Colombo et al., 2024). We hypothesize that known modifiable risk factors for dementia and performance in SPACE will be associated with general cognitive abilities assessed by the MoCA. In contrast, large-scale navigation tasks in SPACE may reflect additional spatial dimensions of cognitive function not captured by the MoCA. We also examine the influence of demographic factors such as age and gender on the various tasks in SPACE and aim to provide normative data across these variables to establish a foundation for future studies.

## Methods

### Participants

**Fig. 1:**
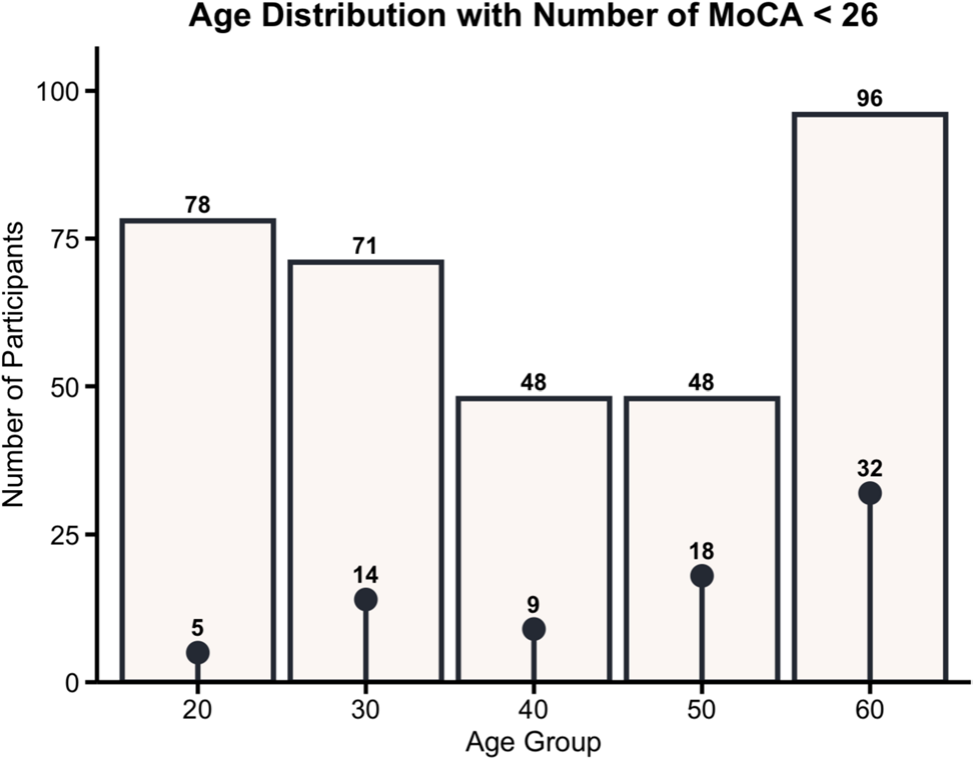
Age distribution and number of participants with MoCA scores < 26. *Bar heights* indicate the number of participants in each age group, and the *overlaid points* show the number of individuals scoring below the MoCA threshold for possible cognitive impairment

We collected data from 348 healthy participants aged 21–76 (M = 45, SD = 16) who were recruited via social media platforms (e.g., Facebook, LinkedIn, Telegram) and community flyers. This sample reflects the age distribution of individuals who were comfortable travelling independently to the Singapore-ETH Centre. Most recruited participants were from Singapore (*n* = 242), and the sample was predominantly Asian (*n* = 273). Only small subsets were from Europe (*n* = 14), North America (*n* = 4), and South America (*n* = 1). Nationality data are missing for 50 participants. Individuals with neurological disorders, severe visual impairment or blindness, deafness, a history of seizures, epilepsy, or recent acute cardiac events were excluded from the study. Due to unforeseen technical issues, such as app crashes or refusal to answer questionnaires, six participants were entirely excluded from all analyses. Additionally, some participants had incomplete data entries, leading to missing values. These incomplete data were omitted from specific analyses, but the participants themselves were not completely excluded. Ultimately, data from 342 participants were included in the final analyses. A subset of this sample overlapped with an earlier SPACE usability study in which different control interfaces (Tap and Anchor conditions), a UI widget (Widget condition), and a simplified configuration of trials and landmarks (Simplified condition) were tested (Colombo et al., 2024). For the present study, all participants who completed the full SPACE protocol were pooled across the testing conditions from the usability study. Figure 1 shows the age distribution and the number of participants who scored < 26 in the MoCA.

Ethical approval for this study was granted by the Parkway Independent Ethics Committee (PIEC/2022/010) and the ETH Zürich Ethics Commission (EK 2021-N-193). Written informed consent was obtained from all participants prior to their involvement in the study. All procedures were conducted in accordance with the Declaration of Helsinki.

### Materials

#### Instruments

Participants completed both the MoCA and SPACE assessments. Before and after these assessments, participants were asked to complete a series of questionnaires, including a digital visual acuity test, a sociodemographic and health questionnaire, the Santa Barbara Sense of Direction scale (Hegarty et al., 2002), a series of usability questionnaires, and a debriefing questionnaire. The digital visual acuity test was based on a Snellen chart and was used only to screen participants for severe visual impairment or blindness. The results from the usability and debriefing questionnaires are detailed in a separate paper (Colombo et al., 2024).

##### MoCA

The MoCA is a widely used cognitive screening tool designed to detect cognitive impairment. The MoCA is a 30-point scale administered in person by a qualified examiner to assess cognitive domains, including memory, executive function, visuospatial skills, language, attention, and orientation. A score below 26 typically indicates MCI. The MoCA has a sensitivity of 90% and a specificity of 87% for predicting cognitive impairment (Nasreddine et al., 2005). In addition, the MoCA (AUC values ranging from 0.71 to 0.99) has better diagnostic accuracy for MCI compared to the MMSE (AUC values ranging from 0.43 to 0.94; Pinto et al., 2019). For detecting AD, the MoCA also outperforms the MMSE, with AUC values ranging from 0.87 to 0.99 for the MoCA and 0.67 to 0.99 for the MMSE.

##### SPACE

**Fig. 2:**
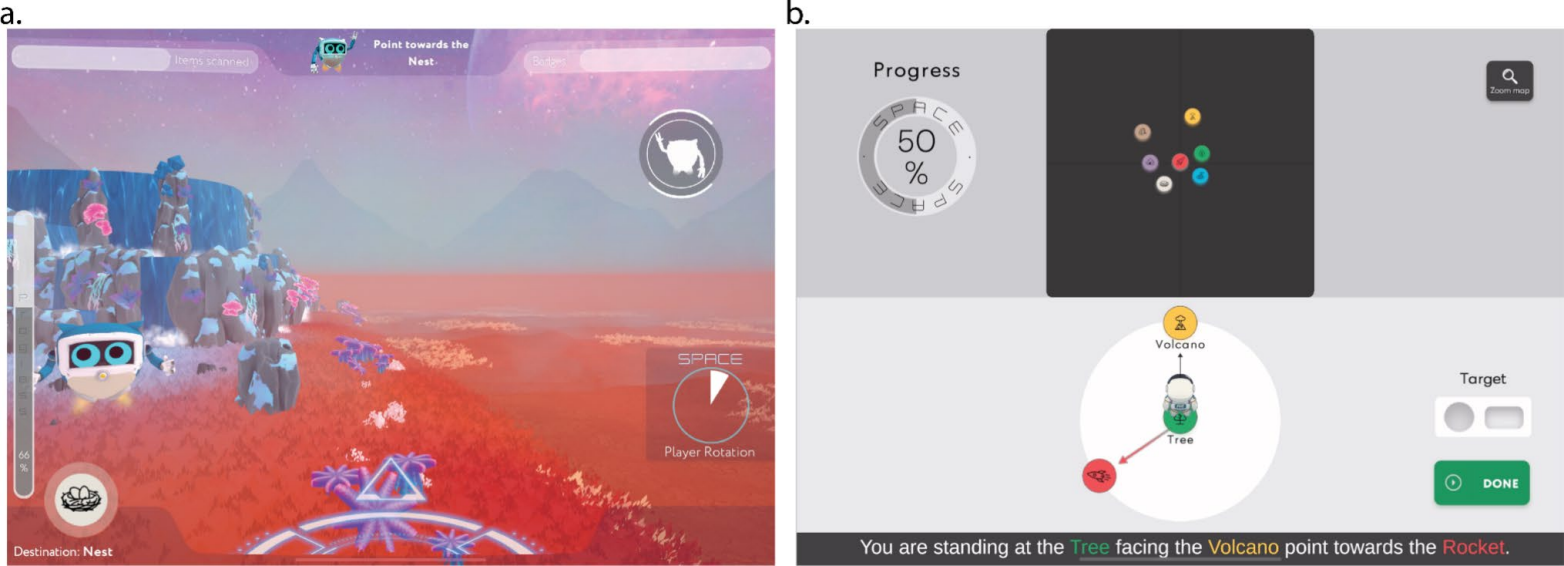
Screenshots from the pointing and perspective-taking tasks in SPACE. **a.** In the pointing task, participants are positioned at a landmark and asked to point toward another landmark encountered during the path integration task. **b.** In the perspective-taking task, participants imagine standing at one landmark and facing another. They must then adjust the target icon to indicate the correct direction to a third landmark from that perspective

SPACE is a novel gamified digital assessment designed to detect deficits in spatial navigation performance that may indicate signs of cognitive impairment (Colombo et al., 2023; Colombo et al., 2024; Colombo et al., 2026; Minta et al., 2026). SPACE is deployed on iPads and includes visuomotor training and five other spatial and navigation tasks. Visuomotor training is critical to minimize learning effects and ensure that performance reflects the ability to control the device (Grübel et al., 2017). In SPACE, participants navigate from a first-person perspective from one landmark to another to learn their relative positions as part of a path integration task. Participants are later probed on their spatial knowledge via pointing (Fig. 2a), mapping, and associative memory tasks. Participants are also asked to complete a perspective-taking task in which they are provided with a top-down representation of the landmarks (Fig. 2b). These tasks are specifically designed to probe the acquisition of spatial knowledge at the environmental scale and are described in Table 1. The virtual environment is intentionally devoid of distinctive features apart from background mountains and the robot that guides participants to the landmarks. Participants encounter only two landmarks per trial and are unable to see a landmark when standing in front of another landmark. The landmarks only become visible when the participant approaches them and fade out when the participant moves away.

**Table 1:** Descriptions of the tasks in SPACE

Visuomotor training	Participants learn to rotate, translate, and integrate these movements by following a robot around the planet from a first-person perspective. In the final training phase, participants are introduced to the logic of the path integration task
Path integration	Participants follow the robot from the rocket to two landmarks, walking along two sides of a triangle from a first-person perspective. At each landmark, the robot scans a different element that will be recalled in a later task. Participants are asked to return unguided to the rocket’s original position, completing the third side of the triangle. Unlike the final training phase, the rocket takes off at the start of each trial and stays invisible until participants signal its landing after completing the trial
Pointing	Participants stand in front of a landmark or the rocket and are asked to complete a series of pointing trials to other landmarks encountered during the path integration task
Mapping	Participants are asked to recreate the configuration of landmarks in the environment they learned by dragging and dropping icons representing the landmarks from a top-down perspective
Associative memory	Participants are presented with a corrected top-down map of the landmarks and are asked to drag and drop icons representing the corresponding elements scanned by the robot during the path integration task
Perspective taking	Participants are provided with the correct top-down map of the environment and are asked to imagine standing at a landmark while facing another landmark. Their task is to indicate the correct bearing toward a third landmark from this perspective

The different experimental conditions (i.e., Tap, Anchor, Widget, and Simplified) have different trial designs for each task. Path integration includes 13 trials in the Tap, Anchor, and Widget conditions, and seven trials in the Simplified condition. The landmark set encountered during path integration forms the basis for all subsequent tasks. The egocentric pointing task includes 15 trials (Tap, Anchor, Widget) or nine trials (Simplified). In the mapping task and associative memory tasks, participants reconstruct the spatial configuration and landmark-item associations of all previously visited landmarks. The perspective-taking task includes 13 trials (Tap, Anchor, Widget) or seven trials (Simplified). A full top-down view of the environment and landmarks, together with a breakdown of the number of participants assigned to each condition, is provided in Supplementary Information A.

##### Sociodemographic and health questionnaire

The sociodemographic and health questionnaire collected information on age, gender, education, background, handedness, tablet experience, and prior navigation training. This questionnaire also collected data on their health status, including vision impairments, chronic conditions, and history of traumatic brain injury, as well as their psychosocial well-being, focusing on levels of depression, anxiety, and stress over the past 6 months. Additionally, the questionnaire addressed health habits, including smoking, alcohol consumption, past-year falls, daily hours of sleep, and weekly hours of walking and vigorous physical activity.

#### Hardware and software

SPACE was deployed on a 10.2-inch iPad with Wi-Fi and 256 GB memory running iOS version 16.6.1. The vision test was conducted using the iPad app MDCalc (https://www.mdcalc.com). All questionnaire data were collected via the Qualtrics XM online survey platform (www.qualtrics.com) on the iPad. Gait data were collected using WitMotion sensors (WT901BLECL Bluetooth 5.0 Accelerometer, https://www.wit-motion.com).

#### Procedure

Before starting the experiment, the experimenter briefed participants on the study’s aim and informed them of their right to take breaks during the session and to withdraw from the experiment at any time without providing a reason. Participants were then asked to read the information sheet and sign the consent form if they agreed to participate. Participants completed the vision test, the MoCA, and the sociodemographic and health questionnaire before playing SPACE. To prevent fatigue, participants were offered the option of a short break prior to starting the SPACE assessment. Each task in SPACE was explained verbally, and additional instructions were displayed within the user interface. Participants took 28 min on average to complete the tasks in SPACE. A detailed breakdown of the time required to complete each task under the various configurations is provided in Supplementary Table A2. After playing SPACE, participants filled out the System Usability Scale (SUS), User Experience Questionnaire (UEQ), NASA Task Load Index (NASA-TLX), Presence questionnaire, and a debriefing questionnaire. To collect gait data, participants walked a circuit for 3 min and then walked the circuit again while counting backwards for 3 min. The gait data were collected for future analyses and are not included in this paper.

#### Analysis

We extracted the following performance variables from the tasks in SPACE. *Visuomotor training performance* was measured as the time (in seconds) required to complete the rotation, translation, circuit, and homing phases. *Path integration distance error* refers to the average distance between the participant’s final position and the target’s original position. Greater distances indicated larger errors. *Egocentric pointing error* was calculated as the average angular deviation (in degrees) between the participant’s estimate and the target landmark. *Mapping accuracy* was assessed using bidimensional regression (Tobler, 1965) to determine the degree of association (*R*^*2*^) between the real map of the environment and the map created by the participant. The *Associative memory score* was computed as the percentage of correct pairings between scanned elements and landmarks. *Perspective taking error* was measured as the average angular deviation (in degrees) between the participant’s estimate and the target landmark. In all analyses, we excluded the associative memory task as an outcome variable due to ceiling effects, which limited variability and prevented the models from converging.

Before conducting inferential statistics, we verified that our data met the assumptions of linear regression. Since some assumptions were violated, we used robust statistics to reduce the influence of outliers on regression estimates by assigning them lower weights in the model-fitting process (Field & Wilcox, 2017). We conducted three robust regression models with the MoCA score as the outcome variable. The first model included only age as a continuous predictor variable and gender as a dichotomous predictor variable. In addition to age and gender, the second model included the eight risk factors for dementia from the sociodemographic and health questionnaire (i.e., education, depression, anxiety, stress, alcohol intake, sleep duration, walking duration, and physical activity duration) as continuous predictor variables. Given that only four participants reported having no formal education, we combined participants with no formal education and those with a high school diploma into a single “lower education” category, contrasting it with participants who have a university-level education. The third model also included the tasks in SPACE (visuomotor, path integration, egocentric pointing, mapping, and perspective taking) as continuous predictors. We compared the first model to the second and the second to the third using robust Wald tests, and assessed differences in fit using changes in *R*^2^. Across all models, missing data were minimal. Models 1 and 2 each excluded one case due to a missing MoCA score, with Model 2 excluding one additional missing value for alcohol consumption. For Models 3, SPACE variables had limited missing values (Pointing: 2; Mapping: 2; Perspective: 10).

Next, we conducted a factor analysis with maximum likelihood extraction and varimax rotation on the MoCA and SPACE scores. Following Dwyer (1937), we also used factor extension to evaluate the loadings of factors not included in the original analysis (i.e., age and gender). Finally, we generated age group and gender norms for each of the tasks in SPACE and visualized the data using continuous norming (Lenhard, Lenhard, Suggate, et al., 2018a, 2018b) across participant ages. All statistical analyses were performed using R Studio Version 2023.06.0 + 421 (R Studio PBC, Boston, MA, http://www.rstudio.com). Robust regressions and the Wald tests were conducted using the R packages *robustbase* (Maechler et al., 2023; Todorov & Filzmoser, 2009) and WRS2 (Mair & Wilcox, 2020). We used the *psych* R package for the factor analysis (Revelle, 2007). Continuous norming was conducted using the *cNORM* R package (Lenhard et al., 2018b). The threshold for significance for all tests was set at α =.05.

## Results

Descriptive statistics for all predictor and outcome variables are listed in Table 2.

**Table 2:** Descriptive statistics for the dementia risk factors and the tasks in SPACE

Descriptive statistics			
Variable categorical	Level		Percentage
Gender	Female Male		56.43 43.57
Education	High school University		27.49 72.51
Variable continuous	Median	Mean	SD
MoCA	27.00	26.68	2.33
Age	43.50	45.13	16.20
Depression	2.00	2.43	1.78
Anxiety	3.00	3.23	2.04
Stress	3.00	3.95	2.23
Alcohol intake	0.00	0.73	2.32
Sleep	7.00	6.75	0.90
Walking	7.00	9.95	9.61
Physical activity	2.00	2.80	2.86
Visuomotor training	241.29	246.14	37.15
Path integration	226.15	246.46	106.80
Pointing	66.45	65.33	20.32
Mapping	0.41	0.46	0.31
Perspective taking	20.64	28.70	23.12

The results of the regression models are presented in Table 3. The first robust regression model, including age and gender, significantly explained 10.7% of the variance in MoCA scores (*χ*^*2*^(2) = 38.278, *p* < 0.001). The results indicated that age has a significant negative effect on MoCA scores (*β* = – 0.27, *p* < 0.001), and males performed significantly worse than females (*β* = – 0.20, *p* = 0.033). The second model, including the individual risk factors as predictors in addition to age and gender, explained an additional 0.2% of the variance in MoCA scores. However, the second model did not significantly explain more variance than the first model (*χ*^*2*^(8) = 9.7729, *p* = 0.281). According to this second model, age (*β* = – 0.24, *p* < 0.001) and gender (*β* = – 0.24, *p* = 0.012) remained significant predictors. None of the individual risk factors significantly affected MoCA scores. The third model, including the scores from the spatial navigation tasks in SPACE, explained an additional 5.2% of the variance, which was a significant improvement over the second model (*χ*^*2*^(5) = 28.129, *p* < 0.001). According to this third model, age (*β* = – 0.12, *p* = 0.044) and gender (*β* = – 0.32, *p* < 0.001) remained significant predictors. Additionally, the pointing (*β* = – 0.12, *p* = 0.031) and perspective-taking (*β* = – 0.17, *p* = 0.007) tasks significantly predicted MoCA scores. We also tested whether SPACE tasks explained variance in MoCA scores beyond age and gender alone by fitting a model with only these predictors. Excluding dementia risk factors did not significantly reduce model fit (see Supplementary Information B1).

**Table 3:** Predictive models of MoCA scores using various risk factors for dementia and tasks from the SPACE assessment

	MoCA		
	Model 1	Model 2	Model 3
	Unstandardized estimates (std. error)		
(Intercept)	28.874 (0.291)***	29.697 (1.149)***	32.205 (1.320)***
Age	– 0.038 (0.007)***	– 0.035 (0.007)***	– 0.017 (0.008)*
Gender_[Male]_	– 0.460 (0.215)*	– 0.561 (0.222)*	– 0.742 (0.222)***
Education_[University]_		0.378 (0.240)	0.201 (0.260)
Depression		0.049 (0.072)	0.034 (0.072)
Anxiety		0.007 (0.090)	0.033 (0.091)
Stress		– 0.005 (0.081)	– 0.019 (0.081)
N Alcohol		0.041 (0.067)	0.031 (0.083)
Sleep		– 0.202 (0.136)	– 0.168 (0.147)
Walking		– 0.013 (0.014)	– 0.015 (0.014)
Physical activity		0.047 (0.034)	0.032 (0.032)
Visuomotor training			– 0.005 (0.003)
Path integration			– 0.001 (0.001)
Pointing			– 0.013 (0.006)*
Mapping			– 0.687 (0.394)
Perspective taking			– 0.017 (0.006)**
No. of Obs	341	340	330
*R*^2^	0.112	0.136	0.199
*R*^2^ Adj	0.107	0.110	0.161
Residual std. error	1.821	1.783	1.711

Model 1 (MoCA ~ Age + Gender); Model 2 (MoCA ~ Age + Gender + Education + Depression + Anxiety + Stress + Alcohol intake + Sleep + Walking + Physical activity); Model 3 (MoCA ~ Age + Gender + Education + Depression + Anxiety + Stress + Alcohol intake + Sleep + Walking + Physical activity + Visuospatial + Path integration + Pointing + Mapping + Perspective taking). * *p* < 0.05; ** *p* < 0.01; *** *p* < 0.001

To confirm that the experimental conditions reported by Colombo and colleagues (2024) did not influence Model 3’s results, the model was reformulated as a linear mixed-effects model with Condition included as a random factor. Including Condition as a random factor did not improve overall model fit and did not substantially change the pattern of significant effects in the models (see Supplementary Information C).

We also explored the extent to which the tasks in SPACE predicted different subdomains of the MoCA (see Supplementary Table D1 in Supplementary Information). We found that path integration (*β* = –.001, *p* <.001), pointing (*β* = – 0.005, *p* =.026), mapping (*β* = – 0.347, *p* =.017), and perspective taking (*β* = – 0.009, *p* <.001) significantly predicted MoCA Visuospatial. Pointing (*β* = – 0.003, *p* =.045) and perspective taking (*β* = – 0.003, *p* =.028) also significantly predicted MoCA Abstraction. In addition, pointing significantly predicted MoCA Attention (*β* = – 0.004, *p* =.004). Furthermore, perspective taking predicted MoCA Naming (*β* = – 0.002, *p* =.011), MoCA Language (*β* = – 0.01, *p* =.040), MoCA Delayed Recall (*β* = – 0.008, *p* =.003), and MoCA Orientation (*β* = – 0.001, *p* =.028).

**Fig. 3:**
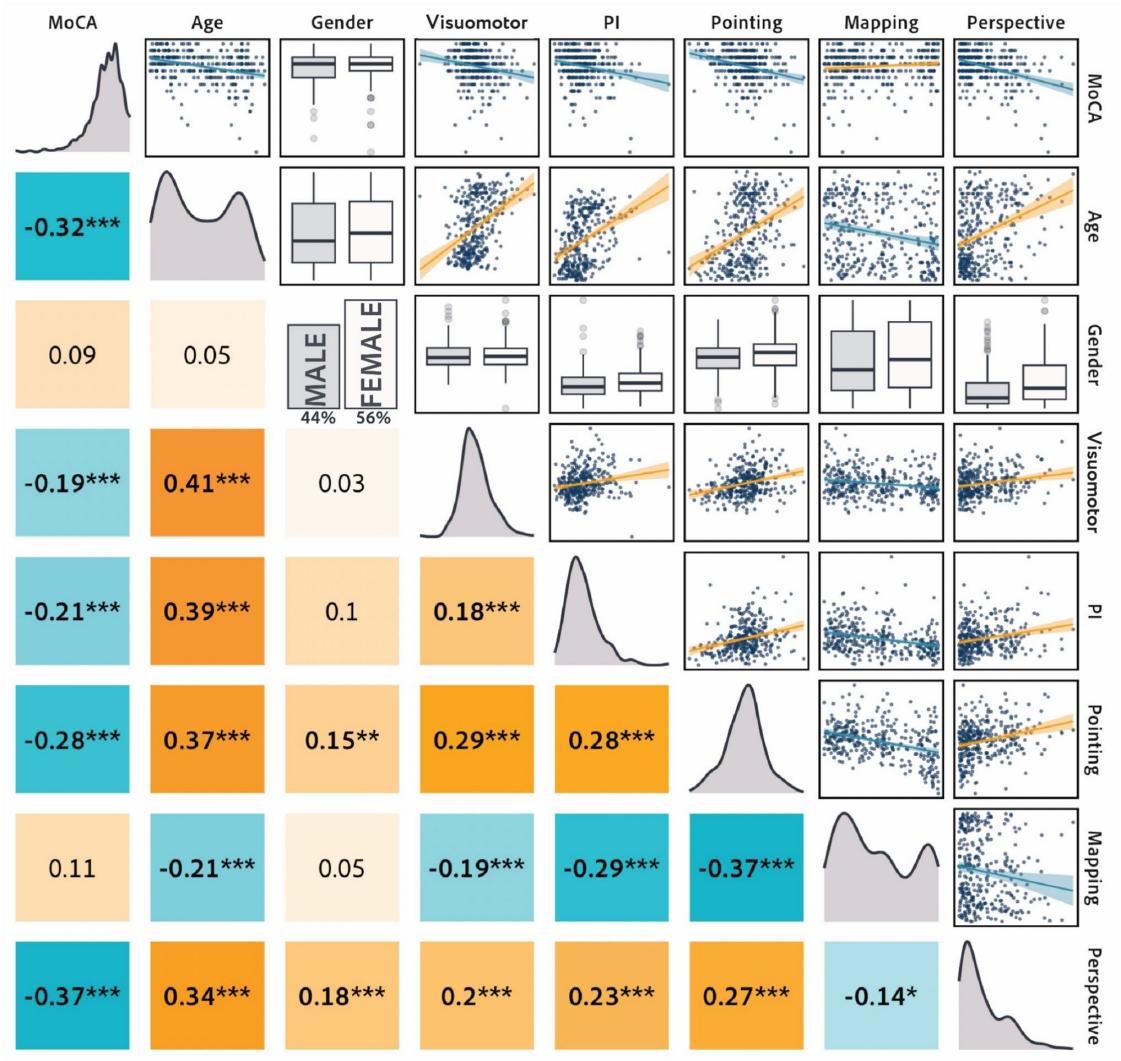
The correlation matrix presents Spearman’s correlation coefficients in the *lower triangle*, density plots along the diagonal illustrating data distributions, and scatterplots in the *upper triangle* showing the relationships between pairs of variables. *Orange* indicates positive correlations, and *blue* represents negative correlations. * *p* < 0.05; ** *p* < 0.01; *** *p* < 0.001

The factor analysis included the variables age, gender, MoCA scores, and the tasks in the SPACE assessment. A correlation analysis revealed several significant relationships, suggesting potential underlying factors that could be extracted from the data (Fig. 3).

**Fig. 4:**
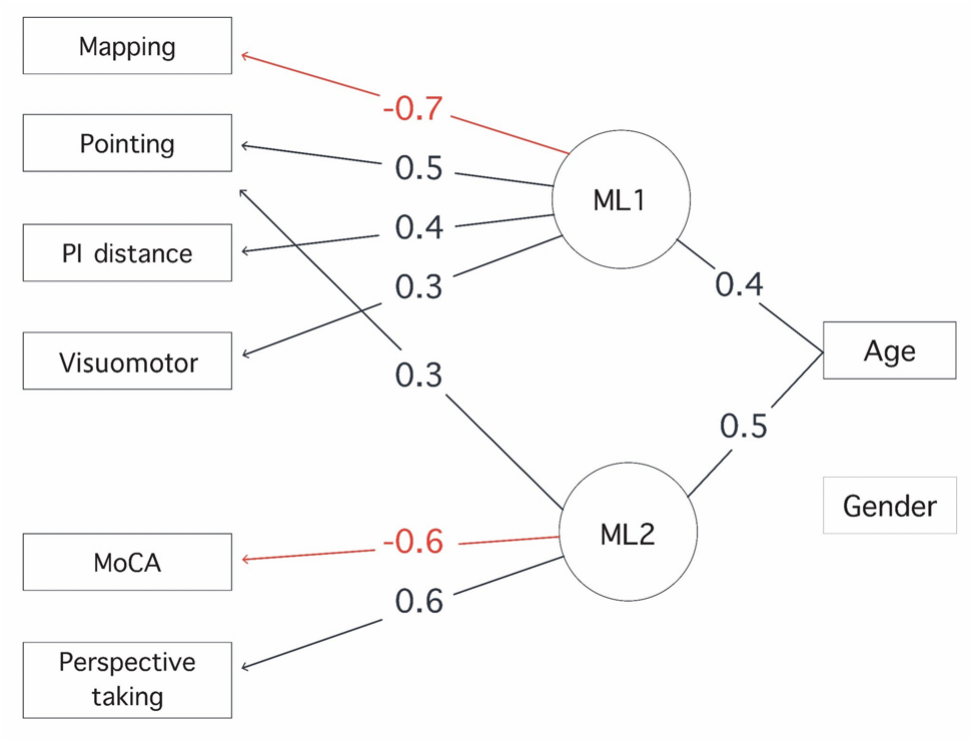
The diagram illustrates the results of the factor analysis for the SPACE tasks and MoCA scores, including the impact of age and gender extensions. Negative loadings are highlighted in *red*

**Fig. 5:**
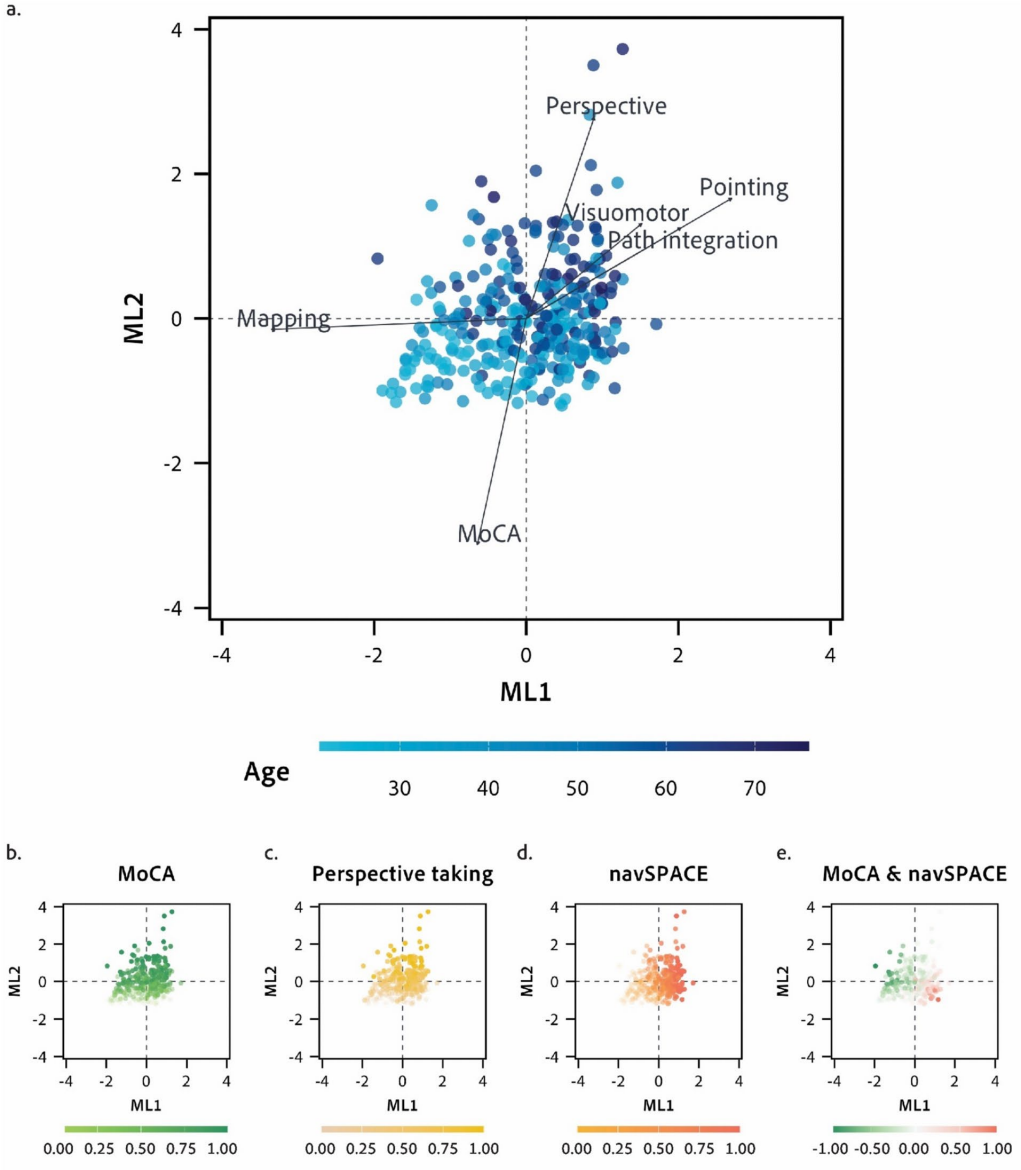
A series of scatter plots for ML1 and ML2, with each *dot* representing an individual participant. **a.** The biplot displays loadings of MoCA and SPACE task scores on extracted factors (ML1 and ML2) and illustrates the relationships among variables. The *length of the arrow* for each variable represents the magnitude of the loading for that variable. The *dots* are colored according to the participants’ ages. **b.** The scatter plot of the factors ML1 and ML2 with dots colored according to MoCA scores (reversed for visualization), along a *light green* (low error) to *dark green* (high error) gradient. **c.** The scatter plot of the factors ML1 and ML2 with dots colored according to the errors on the perspective-taking task, along a *light yellow* (low error) to *dark yellow* (high error) gradient. **d.** The scatter plot of the factors ML1 and ML2, with dots colored according to errors from the navigation tasks in SPACE, along a *light orange* (low error) to *dark orange* (high error) gradient. **e.** The scatter plot of the factors ML1 and ML2 with dots colored according to the difference between reversed MoCA scores and errors from the navigation tasks in SPACE along a gradient from *green* (difference favoring MoCA) to *red* (difference favoring the navigation tasks in SPACE). The visualization demonstrates similar patterns for the MoCA and the perspective-taking task. In addition, there are regions of the biplot that represent participants who performed worse on the navigation tasks in SPACE despite performing well on the MoCA. At the same time, some participants performed worse on the MoCA despite performing well on the navigation tasks in SPACE

Eigenvalues and a parallel analysis indicated the retention of two factors for the factor analysis (Fig. 4). The eigenvalue for the first factor was 2.66, and the eigenvalue for the second factor was 1.10, indicating that these two factors together accounted for 23% of the total variance in the data (Fig. 5). Specifically, ML1 and ML2 explained 8% and 15% of the total variance, respectively. ML1 had a moderate positive loading on visuomotor training (λ = 0.30), stronger positive loadings on pointing error (λ = 0.54) and path integration error (λ = 0.41) tasks, and a negative loading on the mapping accuracy (λ = – 0.67). This factor appears to capture spatial and navigational abilities. ML2 had a strong positive loading on the perspective taking error (λ = 0.56) and a negative loading on MoCA scores (λ = – 0.63), suggesting that this factor is related to cognitive and perceptual abilities (Fig. 3). The standardized loadings for age and gender on ML1 and ML2 showed that age had moderate loadings on both factors (ML1:λ = 0.41, ML2: λ = 0.53), while gender had no impact (ML1: λ = 0.04, ML2: λ = 0.11). For the results of a factor analysis that included the risk factors, see Supplementary Table E1 in the Supplementary Information E.

**Fig. 6:**
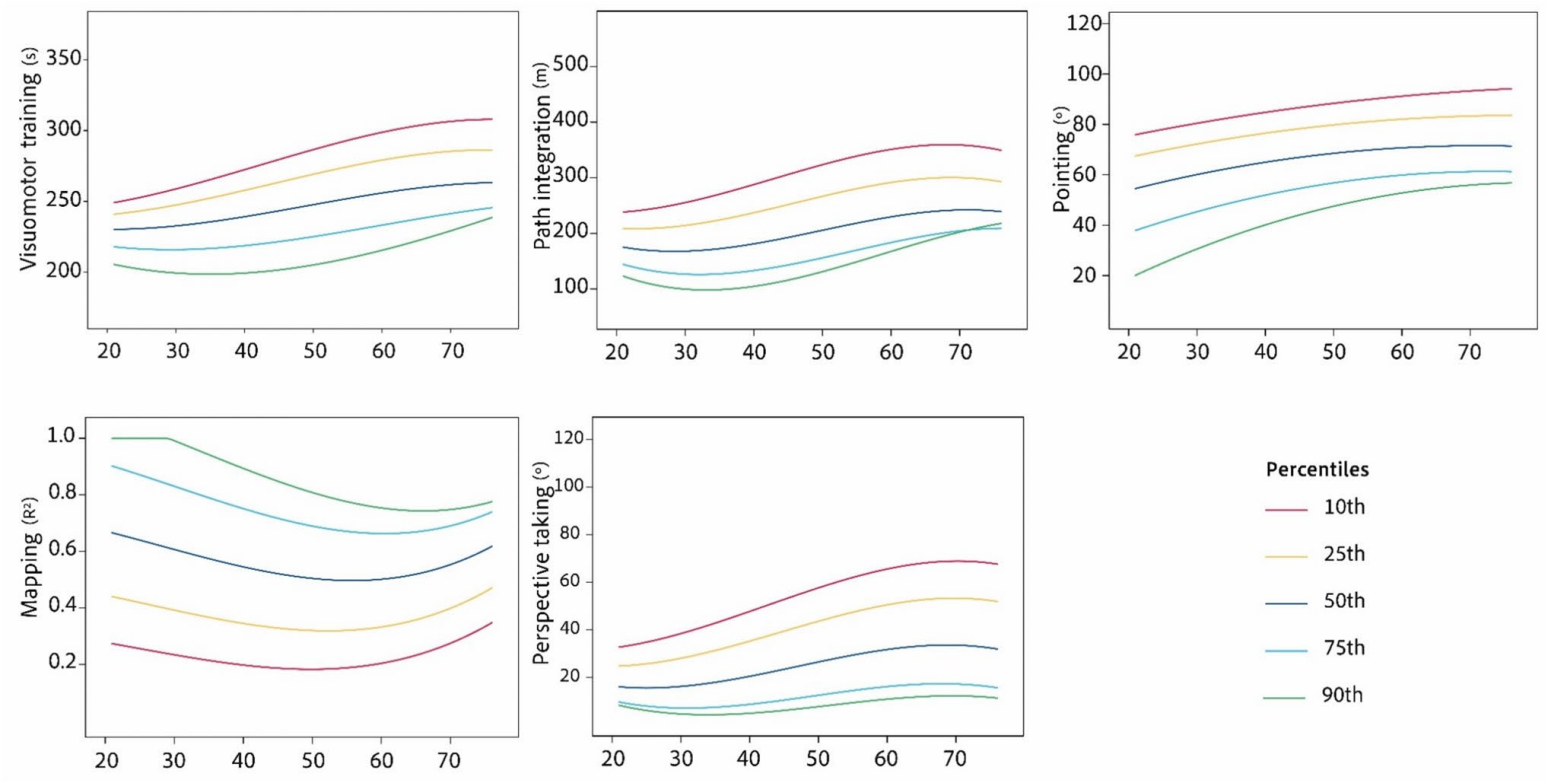
Age norms for the different tasks in SPACE. In the plots, error measures are used for visuomotor training, path integration, egocentric pointing, and perspective taking. The mapping task, however, assesses performance using an accuracy score

To facilitate the application of SPACE for detecting cognitive impairment, we computed age- and gender-specific norms (Table 4) for each SPACE task. Age norms are also listed by age group (i.e., 20–29, 30–39, 40–49, 50–59, and 60 +) and visualized using continuous norming in Fig. 6.

**Table 4:** Normative data for age group and gender

Variable	Age	Gender	Mean	SD	10th Percentile	25th Percentile	50th Percentile	75th Percentile	90th Percentile
Visuomotor training	21–29	Male	223.02	27.62	187.97	204.86	221.79	233.29	258.38
		Female	231.23	24.14	205.24	217.80	227.61	248.50	260.22
		Overall	227.18	26.07	195.35	213.39	224.97	244.32	260.41
	30–39	Male	236.18	24.44	208.60	222.92	236.15	248.79	264.41
		Female	234.00	22.05	209.28	217.90	238.05	251.82	262.23
		Overall	234.95	22.97	208.24	218.43	236.16	250.18	264.13
	40–49	Male	237.26	32.37	195.07	220.85	234.60	260.95	282.28
		Female	245.43	31.40	210.26	223.64	245.22	266.80	285.53
		Overall	241.26	31.82	203.86	222.35	242.96	261.84	286.21
	50–59	Male	251.09	28.54	221.58	231.31	241.57	266.98	291.71
		Female	265.12	37.96	220.60	227.87	265.99	297.24	311.72
		Overall	260.15	35.26	220.05	228.42	260.64	288.98	308.13
	60 +	Male	273.14	41.81	230.12	250.39	269.86	291.21	328.28
		Female	261.51	42.78	218.58	233.96	248.96	291.14	321.88
		Overall	266.33	42.55	220.07	239.79	258.38	291.53	324.45
Path integration	21–29	Male	189.62	67.10	96.06	152.43	182.89	226.77	267.45
		Female	197.79	61.66	119.15	154.22	199.66	230.67	267.63
		Overall	193.76	64.09	109.94	153.20	192.08	227.29	268.19
	30–39	Male	211.51	97.70	110.41	130.93	210.73	259.99	334.77
		Female	213.25	70.61	122.57	155.71	216.40	263.91	299.35
		Overall	212.49	82.81	114.27	144.91	212.32	263.62	315.49
	40–49	Male	197.38	70.17	124.10	153.40	172.20	235.61	305.64
		Female	263.63	116.47	133.58	190.72	222.13	360.43	445.05
		Overall	229.80	100.35	125.75	157.00	201.86	270.33	391.42
	50–59	Male	220.41	81.35	154.05	191.27	202.29	249.72	301.16
		Female	301.60	106.78	183.39	205.65	289.96	385.79	449.29
		Overall	272.85	105.21	173.15	196.61	247.11	348.96	438.03
	60 +	Male	296.39	92.69	180.02	239.59	306.53	350.25	409.11
		Female	301.27	105.95	175.85	230.78	287.24	348.80	441.17
		Overall	299.25	100.17	179.26	232.78	291.64	350.42	427.16
Pointing	21–29	Male	52.95	20.80	24.66	44.49	51.76	67.07	75.54
		Female	56.92	18.73	35.49	41.92	57.44	72.20	78.98
		Overall	54.96	19.74	29.77	42.67	52.36	68.87	76.97
	30–39	Male	59.71	18.43	30.97	50.19	65.14	72.14	81.57
		Female	57.65	20.15	30.92	44.23	60.45	69.98	77.19
		Overall	58.55	19.31	30.92	48.51	61.96	70.30	79.29
	40–49	Male	65.67	18.49	43.00	55.89	66.03	81.66	87.22
		Female	68.57	18.45	50.54	58.75	69.53	78.14	96.79
		Overall	67.09	18.33	43.77	56.43	66.34	80.29	89.78
	50–59	Male	64.49	14.61	47.51	56.76	65.92	74.92	77.30
		Female	75.82	18.05	51.24	66.34	74.02	86.39	100.82
		Overall	71.81	17.62	51.04	61.81	72.16	79.04	94.07
	60 +	Male	68.58	12.03	56.00	61.34	66.66	76.26	83.91
		Female	77.97	15.86	57.91	67.02	75.71	86.55	101.21
		Overall	74.14	15.08	56.69	64.31	73.21	82.43	96.99
Mapping	21–29	Male	0.51	0.33	0.94	0.88	0.50	0.20	0.11
		Female	0.58	0.31	0.96	0.87	0.59	0.26	0.16
		Overall	0.54	0.32	0.95	0.87	0.53	0.22	0.14
	30–39	Male	0.48	0.33	0.95	0.83	0.49	0.20	0.10
		Female	0.55	0.33	0.95	0.85	0.53	0.28	0.12
		Overall	0.52	0.33	0.95	0.86	0.51	0.24	0.11
	40–49	Male	0.52	0.36	0.95	0.89	0.56	0.20	0.10
		Female	0.44	0.31	0.92	0.62	0.40	0.17	0.12
		Overall	0.48	0.33	0.95	0.83	0.41	0.17	0.11
	50–59	Male	0.39	0.30	0.88	0.51	0.28	0.19	0.10
		Female	0.35	0.28	0.87	0.51	0.26	0.12	0.07
		Overall	0.36	0.29	0.88	0.51	0.28	0.13	0.08
	60 +	Male	0.31	0.25	0.68	0.51	0.23	0.10	0.04
		Female	0.43	0.29	0.89	0.62	0.34	0.18	0.08
		Overall	0.38	0.28	0.82	0.56	0.29	0.15	0.06
Perspective taking	21–29	Male	13.30	10.26	4.54	5.85	10.41	18.92	23.91
		Female	22.76	14.04	8.97	11.52	20.14	30.19	43.36
		Overall	18.16	13.15	5.65	8.82	14.59	24.02	33.44
	30–39	Male	22.74	20.15	5.26	8.53	12.96	35.88	51.62
		Female	21.31	16.61	6.25	9.12	14.61	30.00	50.78
		Overall	21.93	18.11	5.56	8.70	13.60	33.76	51.36
	40–49	Male	23.89	25.98	6.45	8.90	13.05	22.72	59.91
		Female	27.55	16.68	7.97	15.66	23.84	37.18	53.07
		Overall	25.76	21.56	6.61	10.54	18.26	36.34	56.47
	50–59	Male	25.27	19.07	5.82	10.31	22.91	32.02	49.12
		Female	34.84	21.57	10.37	17.42	32.36	51.59	64.41
		Overall	31.58	21.05	7.43	14.62	25.49	47.41	61.25
	60 +	Male	33.46	24.70	10.92	14.93	22.90	48.07	69.29
		Female	44.30	26.55	12.87	24.94	39.26	62.08	73.83
		Overall	39.80	26.21	11.53	19.38	33.07	60.17	73.00

## Discussion

This study investigates how dementia risk factors and performance on the spatial navigation tasks in SPACE predict MoCA scores as an indicator of cognitive impairment. The results of our regression analysis revealed that the pointing and perspective taking tasks in SPACE contributed to the prediction of MoCA scores beyond age and gender. Despite previously established relationships between modifiable risk factors and cognitive impairment (Dale et al., 2018; Del Brutto et al., 2015; Livingston et al., 2024), none of the modifiable risk factors in our sample were significant predictors of MoCA scores. Our exploratory factor analysis further revealed that MoCA scores and performance on the perspective taking task in SPACE were associated with a separate factor, distinct from the four navigation-related tasks in SPACE. In addition, we identified two clusters of participants who either performed well on the MoCA and poorly on the navigation tasks or well on the navigation tasks and poorly on the MoCA, suggesting that cognitive assessments may benefit from the combination of MoCA and navigation tasks. We argue that incorporating spatial navigation assessments into cognitive screening tests may improve their sensitivity and offer a more comprehensive and accurate evaluation of cognitive functioning.

A hallmark of MCI and AD is damage to the entorhinal cortex and hippocampus caused by the excessive accumulation of the β-amyloid peptides into neuritic plaques and an abnormal form of the protein tau into neurofibrillary tangles (Jack et al., 2010, 2024). Since structures in the medial temporal lobe (MTL) are often associated with working memory and long-term declarative memory (Bird & Burgess, 2008; Eichenbaum, 2001; Squire, 1992), tasks such as the digit span task and delayed recall are typically used to detect cognitive impairment (Julayanont & Nasreddine, 2017). Indeed, the MoCA includes the digit span task and delayed recall, as well as visuospatial tasks such as trail-making and cube drawing (Julayanont & Nasreddine, 2017; Nasreddine et al., 2005). While both visuospatial tasks require patients to reconstruct small-scale spatial relations, these tasks do not involve the same scale and complexity of navigation skills associated with the MTL. Similar to how performance on declarative memory tasks is used to identify declarative memory impairment, performance on navigation tasks can contribute to the detection of the impairment of spatial skills that rely on the MTL but that are not yet assessed by the MoCA.

SPACE includes various navigation tasks such as visuomotor training, path integration, pointing, and mapping. There is now substantial evidence that performance on each of these individual tasks is associated with age (Lester et al., 2017; Stangl et al., 2020) and can discriminate with varying accuracy between healthy, MCI, and AD patients (deIpolyi et al., 2007; Howett et al., 2019; Mitolo et al., 2013; Segen et al., 2022; Tu et al., 2015). For example, Howett and colleagues (2019) tested healthy and MCI patients in an immersive path integration task and found that path integration error could discriminate between healthy participants and patients with MCI, especially for biomarker-positive patients (CSF amyloid-β and total tau). Notably, the ability of the path integration tasks to discriminate between biomarker-positive and biomarker-negative patients was significantly higher than the Trail Making Test-B and the Four Mountains Test. In addition, deIpolyi and colleagues (2007) found that, although MCI and mild AD patients could recognize landmarks along a learned route, these patients could not accurately identify landmark locations from a map or draw a map of the route. Similar to Howett and colleagues (2019), performance on a neuropsychological assessment, including the MMSE and measures of working memory and visuospatial memory, did not discriminate between patients with and without spatial impairments (deIpolyi et al., 2007). Comparable results were found in a virtual supermarket test in which participants were asked to orient to different goal locations after learning a route (2015). In that study, researchers found that the scores from this spatial orientation task discriminate between control, AD, and frontotemporal dementia participants. Together, this research suggests that spatial navigation assessments can complement traditional screenings for cognitive impairment and may support clinical decision-making.

While previous research focused on patients who had already been clinically diagnosed as cognitively impaired, we tested participants without a diagnosis and with a wide range of ages. We found that performance on the pointing and perspective taking tasks in SPACE predicted MoCA scores. Similarly, Tinella and colleagues (Tinella et al., 2022) found a significant correlation between MoCA scores and a perspective taking task of the same format (Kozhevnikov et al., 2001) for a large sample with a wide range of ages. Interestingly, this relationship was not found in other studies (Muffato & De Beni, 2020; Muffato et al., 2021) with a higher cutoff for MoCA scores (22 instead of 17), suggesting that perspective taking may be useful for discriminating between patients with different levels of cognitive impairment. Indeed, researchers have found that perspective taking tasks can discriminate between healthy and MCI participants (Laczó et al., 2021), healthy and AD participants (Chan et al., 2016; Laczó et al., 2021), and MCI and AD participants (Chan et al., 2016). Researchers have employed several variations of the perspective taking task, which have demonstrated good construct validity (Brucato et al., 2023). As part of SPACE, we introduce a variation of the perspective taking task that predicts MoCA scores and may be more scalable for broader deployment and unsupervised early screening (Tian et al., 2025).

Furthermore, we explored the factor structure underlying the MoCA and the SPACE tasks, revealing two distinct factors. The first factor included the MoCA and the perspective taking task, reinforcing the notion that the perspective taking task in SPACE could capture similar aspects of cognitive function assessed by the MoCA. The second factor combined the locomotion and wayfinding aspects of navigation tasks in SPACE (i.e., visuomotor training, path integration, pointing, and mapping) and may represent an overlooked dimension of cognitive functioning not captured by existing cognitive assessments. This factor structure is consistent with the findings of Meneghetti and colleagues (Meneghetti et al., 2021), who employed confirmatory factor analysis to derive the structure underlying visuospatial tasks and multiple wayfinding-related questionnaires. After finding that wayfinding inclinations (as derived from the questionnaires) underlie a separate factor from the visuospatial tasks, they showed that both factors predicted performance on navigation tasks in VR. Critically, the authors suggested that wayfinding inclinations predicted navigation recall performance because participants were asked to consider space at a larger scale.

Our results are supported by previous work that suggests a separation between small-scale and large-scale spatial abilities. For example, Meneghetti and colleagues (2014) found that small-scale perspective taking abilities cluster separately from large-scale navigation skills and exhibit stronger associations with general cognitive measures. Similarly, Malanchini and colleagues (2020) demonstrated that large-scale navigation abilities form a distinct, coherent factor from other spatial skills. In contrast, Hegarty and colleagues (Hegarty et al., 2006) showed that small-scale spatial tasks, such as mental rotation, are more strongly related to large-scale tasks in visual media than to learning in a real-world environment. However, they also found that a perspective taking task involving four objects was not related to tasks in either visual media or the real environment. Together, these findings suggest that perspective taking and large-scale navigation tasks represent different aspects of cognitive abilities. Indeed, while many of our participants performed either well or poorly on both SPACE and the MoCA, a substantial portion performed well on either SPACE or the MoCA. The present study provides evidence to support the claim that both types of spatial tasks may be used to complement existing screenings for cognitive impairment.

Visuomotor training was the only task in SPACE that did not predict the visuospatial subdomain of MoCA. This result may suggest that visuomotor training performance primarily reflects participants’ ability to locomote using the control interface and captures aspects of fine motor control, rate of learning, and reaction time, rather than large-scale spatial navigation. While these measures do not directly assess spatial abilities, they are nonetheless informative, as the rate of learning (Fernandez-Ballesteros et al., 2005; Wang et al., 2013) and motor performance (Li et al., 2024; Rudd et al., 2023) have been shown to be sensitive to subtle cognitive changes. For example, Fernández-Ballesteros and colleagues (2005) employed a dynamic assessment approach and found that individuals with MCI or mild AD exhibited reduced learning potential, as indicated by their diminished capacity to improve on cognitive tasks compared to that of healthy older adults. Recently, Li and colleagues (2024) employed a tablet-based “drawing and dragging” motor task, which achieved an accuracy of up to 85% in distinguishing individuals with MCI from healthy controls. In their study, older adults with MCI took significantly longer to switch between strokes, showed slower dragging speeds, and obtained lower overall task scores than cognitively healthy peers. In the present study, we may not have found a relationship between visuomotor training and MoCA scores because our sample was composed of healthy adults. However, our own research with clinical populations has shown that visuomotor training reliably predicts MoCA scores (Colombo et al., 2026). Including visuomotor metrics alongside traditional navigation tasks may therefore provide additional insights into early variations in cognitive functioning.

Our factor analysis extension also revealed an effect of age on both factors (but no relationship with gender). According to previous research, spatial memory and related abilities tend to decline with age due to altered computations, functional deficits, and navigational impairments (Lester et al., 2017; Zancada-Menendez et al., 2016). As expected, our regression model revealed that older participants scored lower on the MoCA (Borland et al., 2017; Engedal et al., 2021; Gonçalves et al., 2023; Larouche et al., 2016; Santangelo et al., 2015; Thomann et al., 2018), and this difference may be more pronounced because of the wide age range in our sample. Our regression analysis also found that men had lower MoCA scores than women, aligning with prior research showing a female advantage in both older (Borland et al., 2017; Engedal et al., 2021; Thomann et al., 2018) and younger populations (Larouche et al., 2016), but see (Santangelo et al., 2015) for no differences and (Gonçalves et al., 2023) for a male advantage. Notably, none of the modifiable risk factors in our second model significantly predicted MoCA scores. Specifically, we did not observe a significant effect of education in our study, despite education often being associated with higher MoCA scores (Borland et al., 2017; Engedal et al., 2021; Gonçalves et al., 2023; Larouche et al., 2016; Santangelo et al., 2015; Thomann et al., 2018). These conflicting results may be attributable to the high education level of our sample, with only four participants without a high school diploma. The null effects for depression, anxiety, and stress may be attributable to a lack of sensitivity in our single-item scales for these conditions, although previous research suggests a high correspondence between single items and established measures such as the Depression, Anxiety, and Stress Scale (Verster et al., 2021) and the Geriatric Depression Scale (McCormack et al., 2011). In our study, neither physical activity nor walking was associated with MoCA scores. While sustained physical activity has been found to protect against cognitive impairment (Iso-Markku et al., 2022), the results are mixed (Greendale et al., 2021; Wilson et al., 2002), and cognitive impairment can instead be the cause of a reduction in physical activity. Although previous studies link healthy lifestyles with a lower risk of dementia (Dhana et al., 2022), we found no association between alcohol consumption and MoCA scores in our sample. These results may be partially explained by the extremely low number of drinkers in our study. In more homogeneous and possibly healthier populations, these risk factors may have a reduced sensitivity to predict cognitive impairment.

The present study has at least three notable limitations. First, because we included younger participants primarily from Singapore, we observed less typical variation in several risk factors, including education. This sample prevented us from providing normative values for education level or geographic region. Second, we did not test participants longitudinally and do not know their eventual cognitive status in old age. Third, we focus on the results of a widely used cognitive assessment (i.e., the MoCA), which is not in itself a diagnostic tool. Clinical diagnoses would require a full neuropsychological exam, including tests for biomarkers of neurodegeneration. Despite these limitations, future work can benefit from incorporating SPACE into cognitive assessments, along with the normative data provided in the present study, for the early differentiation of healthy and pathological trajectories. Additionally, we plan to focus on more targeted groups by increasing recruitment of healthy and clinical middle-aged and older adults to strengthen the normative dataset and improve sensitivity to early cognitive changes over a longer period of time. Notably, these tests will include biomarkers as part of a diagnostic tool to evaluate the utility of SPACE further.

## Conclusion

Digital assessments are becoming increasingly popular for assessing cognitive impairments (Berron, Olsson, et al., 2024a, 2024b; Liu et al., 2024; Meier et al., 2021; Thompson et al., 2023). SPACE differs from traditional cognitive assessments by providing spatial tasks in large and complex environments rather than small-scale tasks and questionnaires that focus solely on visuospatial skills, attention, and memory. Here, we show that SPACE predicts MoCA scores beyond traditional demographic and risk factors and has the potential to complement traditional tests for cognitive impairment.

## Supplementary Information

Below is the link to the electronic supplementary material.

Supplementary file1 (PDF 108 kb)

## Data Availability

The data are not publicly available due to ethical constraints. Due to this limitation, the analysis code is illustrated using a synthetic dataset, which enables readers to verify the correctness of their implementation. The synthetic dataset can be found at the following link: https://osf.io/hyabv/?view_only=0eec51e300e14fdfba287a8e52a8f513.

## Code availability

The code used for the analysis can be found at: https://osf.io/hyabv/?view_only=0eec51e300e14fdfba287a8e52a8f513.

## References

[CR1] Alzheimer’s Association. (2024). 2024 Alzheimer’s disease facts and figures. *Alzheimer’s & Dementia: The Journal of the Alzheimer’s Association*, *20*(5), 3708–3821. 10.1002/alz.13809 PMC11095490 38689398

[CR2] *Alzheimer’s Disease Fact Sheet*. (2023, April 5). National Institute on Aging. https://www.nia.nih.gov/health/alzheimers-and-dementia/alzheimers-disease-fact-sheet

[CR3] Barthélemy, N. R., Li, Y., Joseph-Mathurin, N., Gordon, B. A., Hassenstab, J., Benzinger, T. L. S., Buckles, V., Fagan, A. M., Perrin, R. J., Goate, A. M., Morris, J. C., Karch, C. M., Xiong, C., Allegri, R., Mendez, P. C., Berman, S. B., Ikeuchi, T., Mori, H., Shimada, H., … Dominantly Inherited Alzheimer Network. (2020). A soluble phosphorylated tau signature links tau, amyloid and the evolution of stages of dominantly inherited Alzheimer’s disease. *Nature Medicine*, *26*(3), 398–407. 10.1038/s41591-020-0781-z PMC7309367 32161412

[CR4] Berron, D., Glanz, W., Clark, L., Basche, K., Grande, X., Güsten, J., Billette, O. V., Hempen, I., Naveed, M. H., Diersch, N., Butryn, M., Spottke, A., Buerger, K., Perneczky, R., Schneider, A., Teipel, S., Wiltfang, J., Johnson, S., Wagner, M., … Düzel, E. (2024). A remote digital memory composite to detect cognitive impairment in memory clinic samples in unsupervised settings using mobile devices. *NPJ Digital Medicine*, *7*(1), 79. 10.1038/s41746-024-00999-9 PMC10965892 38532080

[CR5] Berron, D., Olsson, E., Andersson, F., Janelidze, S., Tideman, P., Düzel, E., Palmqvist, S., Stomrud, E., & Hansson, O. (2024b). Remote and unsupervised digital memory assessments can reliably detect cognitive impairment in Alzheimer’s disease. *Alzheimer’s & Dementia : The Journal of the Alzheimer’s Association,**20*(7), 4775–4791. 10.1002/alz.13919 PMC11247711 38867417

[CR6] Bird, C. M., & Burgess, N. (2008). The hippocampus and memory: Insights from spatial processing. *Nature Reviews. Neuroscience,**9*(3), 182–194. 18270514 10.1038/nrn2335

[CR7] Borland, E., Nägga, K., Nilsson, P. M., Minthon, L., Nilsson, E. D., & Palmqvist, S. (2017). The Montreal Cognitive Assessment: Normative data from a large Swedish population-based cohort. *Journal of Alzheimer’s Disease : JAD,**59*(3), 893–901. 28697562 10.3233/JAD-170203 PMC5545909

[CR8] Brucato, M., Frick, A., Pichelmann, S., Nazareth, A., & Newcombe, N. S. (2023). Measuring spatial perspective taking: Analysis of four measures using item response theory. *Topics in Cognitive Science,**15*(1), 46–74. 35032360 10.1111/tops.12597

[CR9] Bugallo-Carrera, C., Dosil-Díaz, C., Pereiro, A. X., Anido-Rifón, L., & Gandoy-Crego, M. (2024). Factors that indicate performance on the MoCA 7.3 in healthy adults over 50 years old. *BMC Geriatrics*, *24*(1), 482. 10.1186/s12877-024-05102-1 PMC11144337 38824525

[CR10] Byers, A. L., & Yaffe, K. (2011). Depression and risk of developing dementia. *Nature Reviews. Neurology,**7*(6), 323–331. 21537355 10.1038/nrneurol.2011.60 PMC3327554

[CR11] Castegnaro, A., Ji, Z., Rudzka, K., Chan, D., & Burgess, N. (2023). Overestimation in angular path integration precedes Alzheimer’s dementia. *Current Biology: CB*. 10.1016/j.cub.2023.09.047 10.1016/j.cub.2023.09.047 PMC10957396 37827151

[CR12] Chan, D., Gallaher, L. M., Moodley, K., Minati, L., Burgess, N., & Hartley, T. (2016). The 4 Mountains Test: A short test of spatial memory with high sensitivity for the diagnosis of pre-dementia Alzheimer’s disease. *Journal of Visualized Experiments: JoVE*, *116*. 10.3791/54454 10.3791/54454 PMC5092189 27768046

[CR13] Chételat, G., Villemagne, V. L., Bourgeat, P., Pike, K. E., Jones, G., Ames, D., Ellis, K. A., Szoeke, C., Martins, R. N., O’Keefe, G. J., Salvado, O., Masters, C. L., Rowe, C. C., Australian Imaging Biomarkers and Lifestyle Research Group. (2010). Relationship between atrophy and β‐amyloid deposition in Alzheimer disease. *Annals of Neurology,**67*(3), 317–324. 20373343 10.1002/ana.21955

[CR14] Colombo, G., & Grübel, J. (2023, April). The spatial performance assessment for cognitive evaluation (space): A novel game for the early detection of cognitive impairment. In *Extended Abstracts of the 2023 CHI Conference on Human Factors in Computing Systems* (pp. 1–6).

[CR15] Colombo, G., Minta, K., Grübel, J., Tai, W. L. E., Hölscher, C., & Schinazi, V. R. (2024). Detecting cognitive impairment through an age-friendly serious game: The development and usability of the Spatial Performance Assessment for Cognitive Evaluation (SPACE). *Computers in Human Behavior,**160*(108349), 108349.

[CR16] Colombo, G., Minta, K., Taylor, W. R., Grübel, J., Chong, E., Chong, J. R., ... & Schinazi, V. R. (2026). Spatial navigation as a digital marker for clinically differentiating cognitive impairment severity. *Communications Medicine*. https://doi.org/10.1038/s43856-026-01484-y. (in press). 10.1038/s43856-026-01484-y PMC13086951 41787066

[CR17] Coughlan, G., Coutrot, A., Khondoker, M., Minihane, A.-M., Spiers, H., & Hornberger, M. (2019). Toward personalized cognitive diagnostics of at-genetic-risk Alzheimer’s disease. *Proceedings of the National Academy of Sciences of the United States of America,**116*(19), 9285–9292. 31015296 10.1073/pnas.1901600116 PMC6511014

[CR18] Coughlan, G., Laczó, J., Hort, J., Minihane, A.-M., & Hornberger, M. (2018). Spatial navigation deficits - Overlooked cognitive marker for preclinical Alzheimer disease? *Nature Reviews. Neurology,**14*(8), 496–506. 29980763 10.1038/s41582-018-0031-x

[CR19] Dale, W., Kotwal, A. A., Shega, J. W., Schumm, L. P., Kern, D. W., Pinto, J. M., Pudelek, K. M., Waite, L. J., & McClintock, M. K. (2018). Cognitive function and its risk factors among older US adults living at home. *Alzheimer Disease and Associated Disorders,**32*(3), 207–213. 29334499 10.1097/WAD.0000000000000241 PMC6728147

[CR20] deIpolyi, A. R., Rankin, K. P., Mucke, L., Miller, B. L., & Gorno-Tempini, M. L. (2007). Spatial cognition and the human navigation network in AD and MCI. *Neurology,**69*(10), 986–997. 17785667 10.1212/01.wnl.0000271376.19515.c6

[CR21] Del Brutto, O. H., Mera, R. M., Del Brutto, V. J., Maestre, G. E., Gardener, H., Zambrano, M., & Wright, C. B. (2015). Influence of depression, anxiety and stress on cognitive performance in community‐dwelling older adults living in rural Ecuador: Results of the Atahualpa Project. *Geriatrics & Gerontology International,**15*(4), 508–514. 25155360 10.1111/ggi.12305 PMC11006020

[CR22] Dhana, K., Franco, O. H., Ritz, E. M., Ford, C. N., Desai, P., Krueger, K. R., Holland, T. M., Dhana, A., Liu, X., Aggarwal, N. T., Evans, D. A., & Rajan, K. B. (2022). Healthy lifestyle and life expectancy with and without Alzheimer’s dementia: Population-based cohort study. *BMJ (Clinical Research Ed.),**377*, e068390. 35418416 10.1136/bmj-2021-068390 PMC9006322

[CR23] Dupuis, K., Pichora-Fuller, M. K., Chasteen, A. L., Marchuk, V., Singh, G., & Smith, S. L. (2015). Effects of hearing and vision impairments on the Montreal Cognitive Assessment. *Neuropsychology, Development, and Cognition. Section B, Aging, Neuropsychology and Cognition*, *22*(4), 413–437. 10.1080/13825585.2014.968084 25325767

[CR24] Dwyer, P. S. (1937). The determination of the factor loadings of a given test from the known factor loadings of other tests. *Psychometrika,**2*(3), 173–178.

[CR25] Eichenbaum, H. (2001). The hippocampus and declarative memory: Cognitive mechanisms and neural codes. *Behavioural Brain Research,**127*(1–2), 199–207. 11718892 10.1016/s0166-4328(01)00365-5

[CR26] Engedal, K., Gjøra, L., Bredholt, T., Thingstad, P., Tangen, G. G., Ernstsen, L., & Selbæk, G. (2021). Sex differences on Montreal Cognitive Assessment and Mini-Mental State Examination scores and the value of self-report of memory problems among community-dwelling people 70 years and above: The HUNT study. *Dementia and Geriatric Cognitive Disorders,**50*(1), 74–84. 34038905 10.1159/000516341

[CR27] Epstein, R. A., Patai, E. Z., Julian, J. B., & Spiers, H. J. (2017). The cognitive map in humans: Spatial navigation and beyond. *Nature Neuroscience,**20*(11), 1504–1513. 29073650 10.1038/nn.4656 PMC6028313

[CR28] Fernandez-Ballesteros, R., Zamarron, M. D., & Tarraga, L. (2005). Learning potential: A new method for assessing cognitive impairment. *International Psychogeriatrics,**17*(1), 119–128. 15945596 10.1017/s1041610205000992

[CR29] Field, A. P., & Wilcox, R. R. (2017). Robust statistical methods: A primer for clinical psychology and experimental psychopathology researchers. *Behaviour Research and Therapy,**98*, 19–38. 28577757 10.1016/j.brat.2017.05.013

[CR30] Folstein, M. F. (1983). The Mini-Mental State Examination. *Archives of General Psychiatry,**40*(7), 812. 6860082 10.1001/archpsyc.1983.01790060110016

[CR31] Freire, A. C. C., Pondé, M. P., Liu, A., & Caron, J. (2017). Anxiety and depression as longitudinal predictors of mild cognitive impairment in older adults. *Canadian Journal of Psychiatry. Revue Canadienne de Psychiatrie*, *62*(5), 343–350. 10.1177/0706743717699175 PMC5459229 28346831

[CR32] Fyhn, M., Molden, S., Witter, M. P., Moser, E. I., & Moser, M.-B. (2004). Spatial representation in the entorhinal cortex. *Science,**305*(5688), 1258–1264. 15333832 10.1126/science.1099901

[CR33] Gonçalves, J., Gerardo, B., Nogueira, J., Afonso, R. M., & Freitas, S. (2023). Montreal Cognitive Assessment (MoCA): An update normative study for the Portuguese population. *Applied Neuropsychology. Adult*, 1–7. 10.1080/23279095.2023.2252949 37708840

[CR34] Greendale, G. A., Han, W., Huang, M., Upchurch, D. M., Karvonen-Gutierrez, C., Avis, N. E., & Karlamangla, A. S. (2021). Longitudinal assessment of physical activity and cognitive outcomes among women at midlife. *JAMA Network Open,**4*(3), e213227. 33787912 10.1001/jamanetworkopen.2021.3227 PMC8013795

[CR35] Grübel, J., Thrash, T., Hölscher, C., & Schinazi, V. R. (2017). Evaluation of a conceptual framework for predicting navigation performance in virtual reality. *PLoS ONE,**12*(9), e0184682. 28915266 10.1371/journal.pone.0184682 PMC5600378

[CR36] Hafting, T., Fyhn, M., Molden, S., Moser, M.-B., & Moser, E. I. (2005). Microstructure of a spatial map in the entorhinal cortex. *Nature,**436*(7052), 801–806. 15965463 10.1038/nature03721

[CR37] Hebert, L. E., Bienias, J. L., Aggarwal, N. T., Wilson, R. S., Bennett, D. A., Shah, R. C., & Evans, D. A. (2010). Change in risk of Alzheimer disease over time. *Neurology,**75*(9), 786–791. 20805524 10.1212/WNL.0b013e3181f0754f PMC2938969

[CR38] Hebert, L. E., Weuve, J., Scherr, P. A., & Evans, D. A. (2013). Alzheimer disease in the United States (2010-2050) estimated using the 2010 census. *Neurology,**80*(19), 1778–1783. 23390181 10.1212/WNL.0b013e31828726f5 PMC3719424

[CR39] Hegarty, M., Montello, D. R., Richardson, A. E., Ishikawa, T., & Lovelace, K. (2006). Spatial abilities at different scales: Individual differences in aptitude-test performance and spatial-layout learning. *Intelligence,**34*(2), 151–176.

[CR40] Hegarty, M., Richardson, A. E., Montello, D. R., Lovelace, K., & Subbiah, I. (2002). Development of a self-report measure of environmental spatial ability. *Intelligence,**30*(5), 425–447.

[CR41] Heymann, D., Stern, Y., Cosentino, S., Tatarina-Nulman, O., Dorrejo, J. N., & Gu, Y. (2016). The association between alcohol use and the progression of Alzheimer’s disease. *Current Alzheimer Research,**13*(12), 1356–1362. 27628432 10.2174/1567205013666160603005035 PMC5526221

[CR42] Hort, J., Laczó, J., Vyhnálek, M., Bojar, M., Bureš, J., & Vlček, K. (2007). Spatial navigation deficit in amnestic mild cognitive impairment. *Proceedings of the National Academy of Sciences,**104*(10), 4042–4047. 10.1073/pnas.0611314104 PMC1820705 17360474

[CR43] Howett, D., Castegnaro, A., Krzywicka, K., Hagman, J., Marchment, D., Henson, R., Rio, M., King, J. A., Burgess, N., & Chan, D. (2019). Differentiation of mild cognitive impairment using an entorhinal cortex-based test of virtual reality navigation. *Brain: A Journal of Neurology*, *142*(6), 1751–1766. 10.1093/brain/awz116 PMC6536917 31121601

[CR44] Iso-Markku, P., Aaltonen, S., Kujala, U. M., Halme, H.-L., Phipps, D., Knittle, K., Vuoksimaa, E., & Waller, K. (2024). Physical activity and cognitive decline among older adults. *JAMA Network Open,**7*(2), e2354285. 38300618 10.1001/jamanetworkopen.2023.54285 PMC10835510

[CR45] Iso-Markku, P., Kujala, U. M., Knittle, K., Polet, J., Vuoksimaa, E., & Waller, K. (2022). Physical activity as a protective factor for dementia and Alzheimer’s disease: Systematic review, meta-analysis and quality assessment of cohort and case–control studies. *British Journal of Sports Medicine,**56*(12), 701–709. 35301183 10.1136/bjsports-2021-104981 PMC9163715

[CR46] Jack, C. R., Jr, Wiste, H. J., Vemuri, P., Weigand, S. D., Senjem, M. L., Zeng, G., Bernstein, M. A., Gunter, J. L., Pankratz, V. S., Aisen, P. S., Weiner, M. W., Petersen, R. C., Shaw, L. M., Trojanowski, J. Q., Knopman, D. S., & Alzheimer’s Disease Neuroimaging Initiative. (2010). Brain beta-amyloid measures and magnetic resonance imaging atrophy both predict time-to-progression from mild cognitive impairment to Alzheimer’s disease. *Brain: A Journal of Neurology*, *133*(11), 3336–3348. 10.1093/brain/awq277 PMC2965425 20935035

[CR47] Jack, C. R., Jr., & Holtzman, D. M. (2013). Biomarker modeling of Alzheimer’s disease. *Neuron,**80*(6), 1347–1358. 24360540 10.1016/j.neuron.2013.12.003 PMC3928967

[CR48] Jack, C. R., Jr., Andrews, J. S., Beach, T. G., Buracchio, T., Dunn, B., Graf, A., Hansson, O., Ho, C., Jagust, W., McDade, E., Molinuevo, J. L., Okonkwo, O. C., Pani, L., Rafii, M. S., Scheltens, P., Siemers, E., Snyder, H. M., Sperling, R., Teunissen, C. E., & Carrillo, M. C. (2024). Revised criteria for diagnosis and staging of Alzheimer’s disease: Alzheimer’s Association Workgroup. *Alzheimer’s & Dementia: The Journal of the Alzheimer’s Association,**20*(8), 5143–5169. 10.1002/alz.13859 PMC11350039 38934362

[CR49] Jia, X., Wang, Z., Huang, F., Su, C., Du, W., Jiang, H., Wang, H., Wang, J., Wang, F., Su, W., Xiao, H., Wang, Y., & Zhang, B. (2021). A comparison of the Mini-Mental State Examination (MMSE) with the Montreal Cognitive Assessment (MoCA) for mild cognitive impairment screening in Chinese middle-aged and older population: A cross-sectional study. *BMC Psychiatry,**21*(1), 485. 34607584 10.1186/s12888-021-03495-6 PMC8489046

[CR50] Julayanont, P., & Nasreddine, Z. S. (2017). Montreal cognitive assessment (MoCA): Concept and clinical review. In *Cognitive Screening Instruments* (pp. 139–195). Springer International Publishing.

[CR51] Kim, D. (2022). Effects of depression on changes in cognitive function in older adults: A fixed-effects model analysis using the Korean Longitudinal Study of Aging (KLoSA). *Alzheimer Disease and Associated Disorders,**36*(4), 319–326. 36219139 10.1097/WAD.0000000000000531 PMC9698135

[CR52] Kozhevnikov, M., & Hegarty, M. (7 2001). A dissociation between object manipulation spatial ability and spatial orientation ability. *Memory & Cognition*, *29*(5), 745–756. 10.3758/bf03200477 11531229

[CR53] Kunz, L., Schröder, T. N., Lee, H., Montag, C., Lachmann, B., Sariyska, R., Reuter, M., Stirnberg, R., Stöcker, T., Messing-Floeter, P. C., Fell, J., Doeller, C. F., & Axmacher, N. (2015). Reduced grid-cell-like representations in adults at genetic risk for Alzheimer’s disease. *Science,**350*(6259), 430–433. 26494756 10.1126/science.aac8128

[CR54] Laczó, M., Wiener, J. M., Kalinova, J., Matuskova, V., Vyhnalek, M., Hort, J., & Laczó, J. (2021). Spatial navigation and visuospatial strategies in typical and atypical aging. *Brain Sciences,**11*(11), 1421. 34827423 10.3390/brainsci11111421 PMC8615446

[CR55] Larouche, E., Tremblay, M.-P., Potvin, O., Laforest, S., Bergeron, D., Laforce, R., Monetta, L., Boucher, L., Tremblay, P., Belleville, S., Lorrain, D., Gagnon, J.-F., Gosselin, N., Castellano, C.-A., Cunnane, S. C., Macoir, J., & Hudon, C. (2016). Normative data for the Montreal Cognitive Assessment in middle-aged and elderly Quebec-French people. *Archives of Clinical Neuropsychology : The Official Journal of the National Academy of Neuropsychologists,**31*(7), 819–826. 27625048 10.1093/arclin/acw076 PMC5088608

[CR56] Lenhard, A., Lenhard, W., & Gary, S. (2018). *CNORM - generating continuous test norms*. Psychometrica. 10.13140/RG.2.2.25821.26082

[CR57] Lenhard, A., Lenhard, W., Suggate, S., & Segerer, R. (2018b). A continuous solution to the norming problem. *Assessment,**25*(1), 112–125. 27371826 10.1177/1073191116656437

[CR58] Lester, A. W., Moffat, S. D., Wiener, J. M., Barnes, C. A., & Wolbers, T. (2017). The aging navigational system. *Neuron,**95*(5), 1019–1035. 28858613 10.1016/j.neuron.2017.06.037 PMC5659315

[CR59] Li, A., Li, J., Chai, J., Wu, W., Chaudhary, S., Zhao, J., & Qiang, Y. (2024). Detection of mild cognitive impairment through hand motor function under digital cognitive test: Mixed methods study. *JMIR Mhealth and Uhealth,**12*, e48777. 38924786 10.2196/48777 PMC11237787

[CR60] Liu, W., Yu, L., Deng, Q., Li, Y., Lu, P., Yang, J., Chen, F., Li, F., Zhou, X., Bergeron, M. F., Ashford, J. W., & Xu, Q. (2024). Toward digitally screening and profiling AD: A GAMLSS approach of MemTrax in China. *Alzheimer’s & Dementia : The Journal of the Alzheimer’s Association,**20*(1), 399–409. 10.1002/alz.13430 PMC10916970 37654085

[CR61] Livingston, G., Huntley, J., Liu, K. Y., Costafreda, S. G., Selbæk, G., Alladi, S., Ames, D., Banerjee, S., Burns, A., Brayne, C., Fox, N. C., Ferri, C. P., Gitlin, L. N., Howard, R., Kales, H. C., Kivimäki, M., Larson, E. B., Nakasujja, N., Rockwood, K., … Mukadam, N. (2024). Dementia prevention, intervention, and care: 2024 report of the Lancet standing Commission. *Lancet*. 10.1016/s0140-6736(24)01296-0 39096926 10.1016/S0140-6736(24)01296-0

[CR62] Livingston, G., Huntley, J., Sommerlad, A., Ames, D., Ballard, C., Banerjee, S., Brayne, C., Burns, A., Cohen-Mansfield, J., Cooper, C., Costafreda, S. G., Dias, A., Fox, N., Gitlin, L. N., Howard, R., Kales, H. C., Kivimäki, M., Larson, E. B., Ogunniyi, A., … Mukadam, N. (2020). Dementia prevention, intervention, and care: 2020 report of the Lancet Commission. *Lancet,**396*(10248), 413–446. 32738937 10.1016/S0140-6736(20)30367-6 PMC7392084

[CR63] Lv, Y., Su, H., Li, R., Yang, Z., Chen, Q., Zhang, D., Liang, S., Hu, C., & Ni, X. (2024). A cross-sectional study of the major risk factor at different levels of cognitive performance within Chinese-origin middle-aged and elderly individuals. *Journal of Affective Disorders,**349*, 377–383. 38199420 10.1016/j.jad.2024.01.069

[CR64] Maechler, M., Rousseeuw, P., Croux, C., Todorov, V., Ruckstuhl, A., Salibian-Barrera, M., Verbeke, T., Koller, M., Conceicao, E. L. T., & Anna di Palma, M. (2023). *robustbase: Basic robust statistics*. http://robustbase.r-forge.r-project.org/

[CR65] Mair, P., & Wilcox, R. (2020). Robust statistical methods in R using the WRS2 package. *Behavior Research Methods,**52*(2), 464–488. 31152384 10.3758/s13428-019-01246-w

[CR66] Malanchini, M., Rimfeld, K., Shakeshaft, N. G., McMillan, A., Schofield, K. L., Rodic, M., Rossi, V., Kovas, Y., Dale, P. S., Tucker-Drob, E. M., & Plomin, R. (2020). Evidence for a unitary structure of spatial cognition beyond general intelligence. *Npj Science of Learning*, *5*(1). 10.1038/s41539-020-0067-8 10.1038/s41539-020-0067-8 PMC7331750 32655883

[CR67] McCormack, B., Boldy, D., Lewin, G., & McCormack, G. R. (2011). Screening for depression among older adults referred to home care services: A single-item depression screener versus the Geriatric Depression Scale. *Home Health Care Management & Practice,**23*(1), 13–19.

[CR68] McNaughton, B. L., Battaglia, F. P., Jensen, O., Moser, E. I., & Moser, M.-B. (2006). Path integration and the neural basis of the “cognitive map.” *Nature Reviews. Neuroscience,**7*(8), 663–678. 16858394 10.1038/nrn1932

[CR69] McSorley, V. E., Bin, Y. S., & Lauderdale, D. S. (2019). Associations of sleep characteristics with cognitive function and decline among older adults. *American Journal of Epidemiology,**188*(6), 1066–1075. 30759177 10.1093/aje/kwz037 PMC6545284

[CR70] Meier, I. B., Buegler, M., Harms, R., Seixas, A., Çöltekin, A., & Tarnanas, I. (2021). Using a Digital Neuro Signature to measure longitudinal individual-level change in Alzheimer’s disease: The Altoida large cohort study. *Npj Digital Medicine*. 10.1038/s41746-021-00470-z 34168269 10.1038/s41746-021-00470-z PMC8225898

[CR71] Meneghetti, C., Borella, E., Muffato, V., Pazzaglia, F., & De Beni, R. (2014). Environment learning from spatial descriptions: The role of perspective and spatial abilities in young and older adults. In *Spatial Cognition IX* (pp. 30–45). Springer International Publishing.

[CR72] Meneghetti, C., Miola, L., Toffalini, E., Pastore, M., & Pazzaglia, F. (2021). Learning from navigation, and tasks assessing its accuracy: The role of visuospatial abilities and wayfinding inclinations. *Journal of Environmental Psychology,**75*, 101614.

[CR73] Minta, K., Colombo, G., Tee, M., Low, M., Grübel, J., Wiener, J., ... & Schinazi, V. R. (2026). SPACE: A novel digital tool for assessing hippocampal structural integrity in older adults. *Scientific Reports*. 10.1038/s41598-026-39628-8 PMC12976057 41680411

[CR74] Mitolo, M., Gardini, S., Fasano, F., Crisi, G., Pelosi, A., Pazzaglia, F., & Caffarra, P. (2013). Visuospatial memory and neuroimaging correlates in mild cognitive impairment. *Journal of Alzheimer’s Disease: JAD,**35*(1), 75–90. 23357899 10.3233/JAD-121288

[CR75] Muffato, V., & De Beni, R. (2020). Path learning from navigation in aging: The role of cognitive functioning and wayfinding inclinations. *Frontiers in Human Neuroscience,**14*, 8. 32047427 10.3389/fnhum.2020.00008 PMC6997341

[CR76] Muffato, V., Miola, L., Pazzaglia, F., & Meneghetti, C. (2021). Map learning in aging individuals: The role of cognitive functioning and visuospatial factors. *Brain Sciences,**11*(8), 1033. 34439652 10.3390/brainsci11081033 PMC8394523

[CR77] Nandi, A., Counts, N., Bröker, J., Malik, S., Chen, S., Han, R., Klusty, J., Seligman, B., Tortorice, D., Vigo, D., & Bloom, D. E. (2024). Cost of care for Alzheimer’s disease and related dementias in the United States: 2016 to 2060. *Npj Aging,**10*(1), 13. 38331952 10.1038/s41514-024-00136-6 PMC10853249

[CR78] Nasreddine, Z. S., Phillips, N. A., Bédirian, V., Charbonneau, S., Whitehead, V., Collin, I., Cummings, J. L., & Chertkow, H. (2005). The Montreal Cognitive Assessment, MoCA: A brief screening tool for mild cognitive impairment. *Journal of the American Geriatrics Society,**53*(4), 695–699. 15817019 10.1111/j.1532-5415.2005.53221.x

[CR79] National Center for Health Statistics. (2021). National Vital Statistics System, Mortality 2018–2021 . In *Mortality 2018–2021*. http://wonder.cdc.gov/ucd-icd10-expanded.html

[CR80] O’Keefe, J., & Nadel, L. (1979). The hippocampus as a cognitive map. *The Behavioral and Brain Sciences,**2*(4), 487–494.

[CR81] Paterniti, S., Verdier-Taillefer, M.-H., Dufouil, C., & Alpérovitch, A. (2002). Depressive symptoms and cognitive decline in elderly people. *British Journal of Psychiatry,**181*(5), 406–410. 10.1192/bjp.181.5.406 12411266

[CR82] Patterson, C. (2018). *World Alzheimer Report 2018*. Alzheimer’s Disease International (ADI).

[CR83] Pinto, T. C. C., Machado, L., Bulgacov, T. M., Rodrigues-Júnior, A. L., Costa, M. L. G., Ximenes, R. C. C., & Sougey, E. B. (2019). Is the Montreal Cognitive Assessment (MoCA) screening superior to the Mini-Mental State Examination (MMSE) in the detection of mild cognitive impairment (MCI) and Alzheimer’s disease (AD) in the elderly? *International Psychogeriatrics / IPA,**31*(4), 491–504. 10.1017/S1041610218001370 30426911

[CR84] Potvin, O., Hudon, C., Dion, M., Grenier, S., & Préville, M. (2011). Anxiety disorders, depressive episodes and cognitive impairment in dementia in community-dwelling older men and women. *International Journal of Geriatric Psychiatry,**26*(10), 1080–1088. 21905102 10.1002/gps.2647

[CR85] Rajan, K. B., Weuve, J., Barnes, L. L., McAninch, E. A., Wilson, R. S., & Evans, D. A. (2021). Population estimate of people with clinical Alzheimer’s disease and mild cognitive impairment in the United States (2020-2060). *Alzheimer’s & Dementia,**17*(12), 1966–1975. 10.1002/alz.12362 PMC9013315 34043283

[CR86] Rekers, S., & Finke, C. (2024). Translating spatial navigation evaluation from experimental to clinical settings: The virtual environments navigation assessment (VIENNA). *Behavior Research Methods,**56*(3), 2033–2048. 37166580 10.3758/s13428-023-02134-0 PMC10991013

[CR87] Revelle, W. (2007). psych: Procedures for Psychological, Psychometric, and Personality Research . In *CRAN: Contributed Packages*. The R Foundation. 10.32614/cran.package.psych

[CR88] Rudd, K. D., Lawler, K., Callisaya, M. L., & Alty, J. (2023). Investigating the associations between upper limb motor function and cognitive impairment: A scoping review. *GeroScience,**45*(6), 3449–3473. 37337026 10.1007/s11357-023-00844-z PMC10643613

[CR89] Santangelo, G., Siciliano, M., Pedone, R., Vitale, C., Falco, F., Bisogno, R., Siano, P., Barone, P., Grossi, D., Santangelo, F., & Trojano, L. (2015). Normative data for the Montreal Cognitive Assessment in an Italian population sample. *Neurological Sciences : Official Journal of the Italian Neurological Society and of the Italian Society of Clinical Neurophysiology,**36*(4), 585–591. 25380622 10.1007/s10072-014-1995-y

[CR90] Schmidt-Hieber, C., & Häusser, M. (2013). Cellular mechanisms of spatial navigation in the medial entorhinal cortex. *Nature Neuroscience,**16*(3), 325–331. 23396102 10.1038/nn.3340

[CR91] Segen, V., Ying, J., Morgan, E., Brandon, M., & Wolbers, T. (2022). Path integration in normal aging and Alzheimer’s disease. *Trends in Cognitive Sciences,**26*(2), 142–158. 34872838 10.1016/j.tics.2021.11.001

[CR92] Squire, L. R. (1992). Declarative and nondeclarative memory: Multiple brain systems supporting learning and memory. *Journal of Cognitive Neuroscience,**4*(3), 232–243. 23964880 10.1162/jocn.1992.4.3.232

[CR93] Stangl, M., Kanitscheider, I., Riemer, M., Fiete, I., & Wolbers, T. (2020). Sources of path integration error in young and aging humans. *Nature Communications,**11*(1), 2626. 32457293 10.1038/s41467-020-15805-9 PMC7250899

[CR94] Thomann, A. E., Goettel, N., Monsch, R. J., Berres, M., Jahn, T., Steiner, L. A., & Monsch, A. U. (2018). The Montreal Cognitive Assessment: Normative data from a German-speaking cohort and comparison with international normative samples. *Journal of Alzheimer’s Disease: JAD,**64*(2), 643–655. 29945351 10.3233/JAD-180080 PMC6027948

[CR95] Thompson, L. I., Kunicki, Z. J., Emrani, S., Strenger, J., De Vito, A. N., Britton, K. J., Dion, C., Harrington, K. D., Roque, N., Salloway, S., Sliwinski, M. J., Correia, S., & Jones, R. N. (2023). Remote and in‐clinic digital cognitive screening tools outperform the MoCA to distinguish cerebral amyloid status among cognitively healthy older adults. *Alzheimer’s & Dementia: Diagnosis, Assessment & Disease Monitoring*. 10.1002/dad2.12500 10.1002/dad2.12500 PMC10680059 38026761

[CR96] Tian, N., Colombo, G., & Schinazi, V. (2025). From play to detection: Mini-SPACE as a serious game for unsupervised Cognitive Impairment screening. In *arXiv [cs.HC]*. 10.48550/ARXIV.2511.12068

[CR97] Tinella, L., Lopez, A., Caffò, A. O., Nardulli, F., Grattagliano, I., & Bosco, A. (2022). What these findings tell us. Reply to Kelly et al. What do these findings tell us? Comment on “tinella et al. Cognitive efficiency and fitness-to-drive along the lifespan: The mediation effect of visuospatial transformations. Brain sci. 2021, 11, 1028.” *Brain Sciences,**12*(2), 178. 35203941 10.3390/brainsci12020178 PMC8870729

[CR98] Tobler, W. R. (1965). Computation of the correspondence of geographical patterns. *Papers in Regional Science: The Journal of the Regional Science Association International,**15*(1), 131–139.

[CR99] Todorov, V., & Filzmoser, P. (2009). An object-oriented framework for robust multivariate analysis. *Journal of Statistical Software,**32*(3), 1–47.

[CR100] Tsai, J.-C., Chen, C.-W., Chu, H., Yang, H.-L., Chung, M.-H., Liao, Y.-M., & Chou, K.-R. (2016). Comparing the sensitivity, specificity, and predictive values of the Montreal Cognitive Assessment and Mini-Mental State Examination when screening people for mild cognitive impairment and dementia in Chinese population. *Archives of Psychiatric Nursing,**30*(4), 486–491. 27455923 10.1016/j.apnu.2016.01.015

[CR101] Tu, S., Wong, S., Hodges, J. R., Irish, M., Piguet, O., & Hornberger, M. (2015). Lost in spatial translation - A novel tool to objectively assess spatial disorientation in Alzheimer’s disease and frontotemporal dementia. *Cortex; a Journal Devoted to the Study of the Nervous System and Behavior,**67*, 83–94. 25913063 10.1016/j.cortex.2015.03.016

[CR102] van der Ham, I. J. M., Claessen, M. H. G., Evers, A. W. M., & van der Kuil, M. N. A. (2020). Large-scale assessment of human navigation ability across the lifespan. *Scientific Reports,**10*(1), 3299. 32094394 10.1038/s41598-020-60302-0 PMC7039892

[CR103] Verster, J. C., Sandalova, E., Garssen, J., & Bruce, G. (2021). The use of single-item ratings versus traditional multiple-item questionnaires to assess mood and health. *European Journal of Investigation in Health Psychology and Education,**11*(1), 183–198. 34542458 10.3390/ejihpe11010015 PMC8314344

[CR104] Wang, P., Li, J., Li, H., & Zhang, S. (2013). Differences in learning rates for item and associative memories between amnestic mild cognitive impairment and healthy controls. *Behavioral and Brain Functions: BBF,**9*(1), 29. 23886305 10.1186/1744-9081-9-29 PMC3751153

[CR105] Weisberg, S. M., Schinazi, V. R., Newcombe, N. S., Shipley, T. F., & Epstein, R. A. (2014). Variations in cognitive maps: Understanding individual differences in navigation. *Journal of Experimental Psychology. Learning, Memory, and Cognition,**40*(3), 669–682. 24364725 10.1037/a0035261

[CR106] Wiener, J. M., Carroll, D., Moeller, S., Bibi, I., Ivanova, D., Allen, P., & Wolbers, T. (2020). A novel virtual-reality-based route-learning test suite: Assessing the effects of cognitive aging on navigation. *Behavior Research Methods,**52*(2), 630–640. 31236900 10.3758/s13428-019-01264-8 PMC7148270

[CR107] Wilson, R. S., Mendes De Leon, C. F., Barnes, L. L., Schneider, J. A., Bienias, J. L., Evans, D. A., & Bennett, D. A. (2002). Participation in cognitively stimulating activities and risk of incident Alzheimer’s disease. *JAMA: The Journal of the American Medical Association,**287*(6), 742–748. 11851541 10.1001/jama.287.6.742

[CR108] World Health Organization (2023, March 15). Dementia. https://www.who.int/news-room/fact-sheets/detail/dementia

[CR109] Xu, G., Liu, X., Yin, Q., Zhu, W., Zhang, R., & Fan, X. (2009). Alcohol consumption and transition of mild cognitive impairment to dementia. *Psychiatry and Clinical Neurosciences,**63*(1), 43–49. 19154211 10.1111/j.1440-1819.2008.01904.x

[CR110] Zancada-Menendez, C., Sampedro-Piquero, P., Lopez, L., & McNamara, T. P. (2016). Age and gender differences in spatial perspective taking. *Aging Clinical and Experimental Research,**28*(2), 289–296. 26138819 10.1007/s40520-015-0399-z

[CR111] Zawar, I., Mattos, M. K., Manning, C., & Quigg, M. (2022). Sleep disturbances, cognitive status, and biomarkers of dementia. *Journal of Alzheimer’s Disease,**89*(4), 1367–1374. 36031904 10.3233/JAD-220664 PMC11218918

