# Supplementary material for: Beyond traditional assessments of cognitive status: Exploring the potential of spatial navigation tasks
Source: Behav Res Methods. 2026 Apr 17;58(5):126. doi: 10.3758/s13428-026-02998-y (PMC13090228; doi:10.3758/s13428-026-02998-y)
Supplement: Supplementary file 1 — Supplementary file1 (PDF 108 kb) [file 13428_2026_2998_MOESM1_ESM.pdf]

Supplementary Materials

Supplementary Information A: Spatial layouts of the landmark configurations in the virtual environment

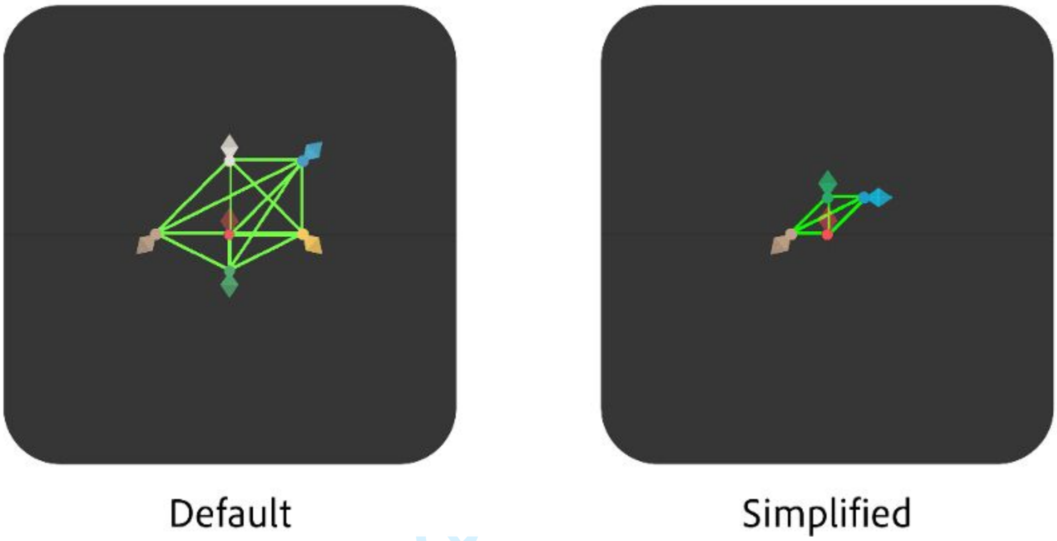

Supplementary Figure A1. Schematic representation of the Default or a Simplified configuration of landmarks in the virtual environment. The red rhombus represents the rocket's position. The other rhombuses represent the location of the other landmarks in the default and simplified configurations.

Proportion of participants across configurations and experimental conditions

This table summarises the proportion of participants who completed the SPACE assessment using either the Default or Simplified configuration of trials, and the corresponding experimental conditions. Overall, 64% of participants used the Default configuration (Tap, Anchor, and Widget), while 36% used the Simplified configuration. Because the Anchor control interface was used in every condition except Tap & Swipe, it was applied across both the Default configuration (Anchor and Widget conditions) and the Simplified configuration. As a result, 73% of participants used the Anchor interface, whereas 27% used Tap & Swipe. The table provides the full breakdown of participants by configuration and control condition.

Supplementary Table A1. Distribution of participants across SPACE configurations and control interface conditions.

Configuration	Condition	N (%)
**Default 220 (64%)**	Tap & Swipe control	92 (27%)
	Anchor control	92 (27%)
	UI Widget	36 (10%)
**Simplified 122 (36%)**	Simplified trials	122 (36%)

Task completion and total SPACE duration across different configurations

To provide a detailed estimate of SPACE administration duration, we report mean completion times for each of the six component tasks (Visuomotor, Path Integration, Pointing, Mapping, Memory, and Perspective Taking) across the two usability configurations used in the study. On average, participants required approximately 36 minutes to complete SPACE in the Default condition and 21 minutes in the Simplified condition.

Supplementary Table A2. Mean task completion times (in seconds) for each SPACE task and total SPACE duration, reported separately for the Default and Simplified conditions. N refers to the number of participants per condition.

Summary of Task Times by Condition

	Means (in seconds)							
Configuration	Visuomotor	Path integration	Pointing	Mapping	Memory	Perspective taking	SPACE Time	N
Default	243.43	1,061.11	355.30	82.13	41.27	448.12	2,194.07	220
Simplified	251.01	472.64	214.87	52.98	23.82	250.82	1,242.32	122

For Review Only

Supplementary Information B: Regression of age, gender and SPACE predicting MoCA scores excluding the risk factors

To examine whether the SPACE tasks provide added explanatory value relative to age and gender, irrespective of dementia risk factors, we compared nested models using robust Wald tests. Adding SPACE measures to the risk-factor model significantly improved model fit (Model 2 → Model 3). Importantly, removing the risk factors from the full model did not significantly reduce model performance (Model 3 → Model 4), $\chi^2(8) = 6.07$, $p = .639$. This result indicates that the dementia risk factors do not meaningfully contribute to variance in MoCA scores once the SPACE tasks are included. Because the risk factor model (Model 2) and the SPACE-only model (Model 4) are not nested, formal model comparison via ANOVA is not applicable. Instead, we compared the models using pseudo-R^2 and RMSE. The SPACE-only model showed higher explanatory power (pseudo-R^2 = 0.167) and lower prediction error (RMSE = 2.06) than the risk-factor model (pseudo-R^2 = 0.110; RMSE = 2.17).

Supplementary Table B1. Robust regression of MoCA scores, including and excluding dementia risk factors.

	Regression predicting MoCA			
	Model 1	Model 2	Model 3	Model 4
(Intercept)	**28.874 (0.291)*** **	**29.697 (1.149)*** **	**32.205 (1.320)*** **	**31.336 (0.834)*** **
Age	**-0.038 (0.007)*** **	**-0.035 (0.007)*** **	**-0.017 (0.008)* **	**-0.020 (0.008)* **
Gender [Male]	**-0.460 (0.215)* **	**-0.561 (0.222)* **	**-0.742 (0.222)*** **	**-0.680 (0.216)** **
Education [University]		0.378 (0.240)	0.201 (0.260)	
Depression		0.049 (0.072)	0.034 (0.072)	
Anxiety		0.007 (0.090)	0.033 (0.091)	
Stress		-0.005 (0.081)	-0.019 (0.081)	
N Alcohol		0.041 (0.067)	0.031 (0.083)	
Sleep		-0.202 (0.136	-0.168 (0.147)	
Walking		-0.013 (0.014)	-0.015 (0.014)	
Physical activity		0.047 (0.034)	0.032 (0.032)	
Visuomotor training			-0.005 (0.003)	-0.005 (0.003)
Path integration			-0.001 (0.001)	-0.001 (0.001)
Pointing			-0.013 (0.006)*	**-0.014 (0.006)* **
Mapping			-0.687 (0.394)	-0.656 (0.375)
Perspective taking			-0.017 (0.006)**	**-0.019 (0.006)** **
Num.Obs.	341	340	330	331
R2	0.112	0.136	0.199	0.185
R2 Adj.	0.107	0.110	0.161	0.167
RMSE	2.21	2.17	2.04	2.06

Note: Model 1: MoCA ~ Age + Gender; Model 2: MoCA ~ Age + Gender + Education + Depression + Anxiety + Stress + Alcohol + Sleep + Walking + Physical Activity + Visuomotor Training; Model 3: MoCA ~ Age + Gender + Education + Depression + Anxiety + Stress + Alcohol + Sleep + Walking + Physical Activity + Visuomotor Training + Path Integration + Pointing + Mapping + Perspective Taking; Model 4: MoCA ~ Path Integration + Pointing + Mapping + Perspective Taking. p < 0.1, * p < 0.05, ** p < 0.01, *** p < 0.001.

For Review Only

Supplementary Information C: Controlling for Experimental Conditions

The study sample in the current manuscript partially overlaps with our previous usability publication on SPACE, but addresses a different research question. Whereas the prior work compared four usability conditions to evaluate interface design and user experience, the present study focuses on individual differences in spatial ability and their relationship with demographic, cognitive, and dementia-relevant risk factors. To maximise statistical power, participants from all usability configurations were pooled and supplemented with 87 additional recruits (total N = 348). Because participants completed the SPACE under different interface conditions, we verified whether these conditions influenced the prediction of MoCA scores by fitting a linear mixed-effects model that included Condition as a random factor. The model fit did not improve relative to the corresponding fixed-effects model (LRT: $\chi^2 = 0.648$, $p = 0.421$), indicating that the interface condition did not contribute meaningfully to MoCA variance. Importantly, the same predictors (gender, pointing, and perspective taking) remained significant in both models. Supplementary Table C1 reports the distribution of participants across interface configurations and control conditions.

Supplementary Table C1. Linear mixed-effects model examining whether interface condition influenced MoCA scores when controlling for demographic variables, risk factors, and SPACE task performance. Condition was included as a random effect and did not significantly improve model fit. Significant predictors (age, gender, pointing, and perspective taking) remained consistent across models.

	MoCA							
Predictors	Estimates	std. Error	std. Beta	standardised std. Error	CI	standardised CI	Statistic	p
(Intercept)	33.00	1.47	0.09	0.12	30.11 – 35.89	-0.15 – 0.33	22.47	**<0.001**
Age	-0.02	0.01	-0.11	0.07	-0.03 – 0.00	-0.24 – 0.02	-1.72	0.086
Gender $_{[Male]}$	-0.97	0.24	-0.42	0.10	-1.45 – -0.49	-0.62 – -0.21	-4.01	**<0.001**
Education $_{[University]}$	0.26	0.27	0.11	0.12	-0.29 – 0.80	-0.12 – 0.34	0.93	0.354
Depression	0.01	0.10	0.01	0.07	-0.18 – 0.20	-0.14 – 0.15	0.11	0.911
Anxiety	-0.00	0.10	-0.00	0.09	-0.20 – 0.19	-0.18 – 0.17	-0.05	0.963
Stress	0.02	0.08	0.02	0.08	-0.14 – 0.19	-0.14 – 0.18	0.28	0.779
N Alcohol	0.04	0.05	0.04	0.05	-0.06 – 0.14	-0.06 – 0.14	0.81	0.420
Sleep	-0.25	0.13	-0.09	0.05	-0.51 – 0.02	-0.19 – 0.01	-1.85	0.065
Walking	-0.02	0.01	-0.10	0.05	-0.05 – 0.00	-0.20 – 0.00	-1.94	0.053
Physical activity	0.04	0.04	0.04	0.05	-0.05 – 0.12	-0.06 – 0.14	0.85	0.397
Visuospatial	-0.00	0.00	-0.03	0.06	-0.01 – 0.01	-0.14 – 0.08	-0.57	0.569
PI distance	-0.00	0.00	-0.08	0.06	-0.00 – 0.00	-0.20 – 0.03	-1.39	0.164
Pointing	-0.02	0.01	-0.21	0.06	-0.04 – -0.01	-0.33 – -0.09	-3.55	**<0.001**
Mapping	-0.70	0.43	-0.10	0.06	-1.54 – 0.13	-0.21 – 0.02	-1.65	0.099
Perspective	-0.03	0.01	-0.28	0.06	-0.04 – -0.02	-0.39 – -0.17	-5.07	**<0.001**
**Random Effects**								
σ^2	4.13							
$\tau_{00\ Condition}$	0.09							
ICC	0.02							
$N_{Condition}$	4							
LRT χ^2 / p value	0.648 / 0.421							
Observations	330							
Marginal R^2 / Conditional R^2	0.260 / 0.275							

For Review Only

Supplementary Information D: Associations Between SPACE Tasks and MoCA Subdomains

To provide a more fine-grained examination of the relationship between SPACE performance and general cognition, we conducted linear regressions with each MoCA subdomain (Visuospatial, Naming, Attention, Language, Abstraction, Delayed Recall, Orientation) as the outcome variable and SPACE task performance as predictors while controlling for age and gender. While the total MoCA score captures broad cognitive functioning, analysing subdomains allows the identification of domain-specific associations. Results (Supplementary Table D1) indicate that MoCA Visuospatial performance was significantly predicted by nearly all SPACE tasks, consistent with the spatial demands of the task. Perspective taking emerged as the most consistent predictor across multiple subdomains, including Naming, Language, Delayed Recall, and Orientation, suggesting it engages broader cognitive processes such as executive function and spatial transformation. Pointing showed more selective associations with Attention and Abstraction.

Supplementary Table D1. Linear regression models examining the association between SPACE task performance and individual MoCA subdomains. The table reports unstandardized regression coefficients (β), standard errors (SE), t-values, and p-values for each predictor across the MoCA subdomains (Visuospatial, Naming, Attention, Language, Abstraction, Delayed Recall, and Orientation). Predictors include demographic variables (age and gender) and SPACE tasks (visuospatial training, path integration, pointing, mapping, and perspective-taking). The model fit (R^2) and sample size are provided for each domain.

MoCA subdomains						
	Predictor	β	(SE)	t value	p value	R^2 / N
**Visuospatial**	Intercept	5.399	(0.338)	15.96	<.001***	0.125 / 331
	Age	0.005	(0.003)	1.46	0.145	
	Gender	-0.214	(0.086)	-2.50	0.013*	
	Visuomotor training	0.000	(0.001)	0.20	0.841	
	Path integration	-0.001	(0.000)	-3.34	<.001***	
	Pointing	-0.005	(0.002)	-2.24	0.026*	
	Mapping	-0.347	(0.145)	-2.39	0.017*	
	Perspective	-0.009	(0.002)	-4.45	<.001***	
**Naming**	Intercept	3.066	(0.141)	21.77	<.001***	0.047 / 331
	Age	-0.001	(0.001)	-0.88	0.378	
	Gender	-0.019	(0.036)	-0.53	0.595	
	Visuomotor training	0.000	(0.001)	0.58	0.560	
	Path integration	-0.000	(0.000)	-0.39	0.697	
	Pointing	-0.001	(0.001)	-1.29	0.197	
	Mapping	-0.009	(0.060)	-0.15	0.880	
	Perspective	-0.002	(0.001)	-2.56	0.011*	
**Attention**	Intercept	6.421	(0.199)	32.24	<.001***	0.067 / 331
	Age	0.000	(0.002)	0.02	0.983	
	Gender	-0.045	(0.050)	-0.90	0.368	
	Visuomotor training	-0.001	(0.001)	-1.01	0.311	
	Path integration	-0.000	(0.000)	-0.81	0.418	
	Pointing	-0.004	(0.001)	-2.86	0.004**	
	Mapping	-0.066	(0.086)	-0.77	0.444	
	Perspective	-0.002	(0.001)	-1.82	0.069	
**Language**	Intercept	2.686	(0.376)	7.14	<.001***	0.145 / 331
	Age	-0.017	(0.004)	-4.86	<.001***	
	Gender	-0.260	(0.095)	-2.73	0.007**	
	Visuomotor training	0.000	(0.001)	0.34	0.734	
	Path integration	0.000	(0.000)	0.82	0.415	

MoCA subdomains						
	Pointing	-0.002	(0.003)	-0.93	0.351	
	Mapping	-0.102	(0.162)	-0.63	0.529	
	Perspective	-0.005	(0.002)	-2.38	0.018*	
**Abstraction**	Intercept	2.042	(0.240)	8.50	<.001***	0.041 / 331
	Age	0.003	(0.002)	1.20	0.230	
	Gender	-0.010	(0.061)	-0.17	0.865	
	Visuomotor training	-0.000	(0.001)	-0.01	0.994	
	Path integration	-0.000	(0.000)	-1.38	0.169	
	Pointing	-0.003	(0.002)	-2.01	0.045*	
	Mapping	-0.115	(0.103)	-1.11	0.266	
	Perspective	-0.003	(0.001)	-2.21	0.028*	
**Delayed recall**	Intercept	5.644	(0.461)	12.24	<.001***	0.125 / 331
	Age	-0.010	(0.004)	-2.35	0.019*	
	Gender	-0.270	(0.117)	-2.31	0.021*	
	Visuomotor training	-0.001	(0.002)	-0.80	0.422	
	Path integration	-0.000	(0.001)	-0.06	0.948	
	Pointing	-0.005	(0.003)	-1.66	0.098	
	Mapping	0.086	(0.198)	0.43	0.665	
	Perspective	-0.008	(0.003)	-3.04	0.003**	
**Orientation**	Intercept	6.004	(0.081)	74.14	<.001***	0.028 / 331
	Age	-0.000	(0.001)	-0.58	0.565	
	Gender	-0.011	(0.021)	-0.54	0.589	
	Visuomotor training	0.000	(0.000)	0.02	0.987	
	Path integration	0.000	(0.000)	1.52	0.130	
	Pointing	-0.000	(0.001)	-0.11	0.911	
	Mapping	-0.042	(0.035)	-1.20	0.233	
	Perspective	-0.001	(0.000)	-2.21	0.028*	

iew Only

Supplementary Information E: Factor analysis of SPACE including dementia risk factors

An exploratory factor analysis with varimax rotation was conducted to examine the underlying structure of cognitive (MoCA), spatial (SPACE tasks), and modifiable dementia risk factors (depression, anxiety, stress, alcohol use, sleep, walking, physical activity). Eigenvalues and parallel analysis supported the retention of three factors (ML1, ML2, ML3), explaining 32.7% of the total variance (ML1 = 16.4%, ML2 = 8.5%, ML3 = 7.8%). ML1 loaded strongly on depression (λ = 0.78), anxiety (λ = 0.93), and stress (λ = 0.80), representing a mental health factor. ML2 showed a moderate negative loading on MoCA (λ = -0.62) and a positive loading on perspective taking (λ = 0.55), reflecting cognitive and perceptual skills. ML3 showed positive loadings on mapping (λ = 0.54) and negative loadings on path integration (λ = -0.38) and pointing (λ = -0.58), capturing spatial and navigation skills. Standardised loadings of age and gender indicated that age had a minor influence on ML1 (λ = -0.24) and a moderate influence on ML2 (λ = 0.50) and ML3 (λ = -0.36), while gender had negligible effects on all factors. Importantly, the modifiable risk factors did not load significantly onto ML2 or ML3, suggesting that the SPACE measures and MoCA capture variance independently of these risk factors.

For Review Only

Supplementary Table E1. Exploratory factor analysis of cognitive, spatial, and dementia risk factors.

Variable	ML1	ML2	ML3
MoCA	–	**−0.62**	–
Visuomotor training	–	0.32	−0.28
Path integration	–	0.28	**−0.38**
Pointing	–	0.35	**−0.58**
Mapping	–	−0.12	**0.54**
Perspective Taking	–	**0.55**	−0.15
Depression	**0.78**	–	0.11
Anxiety	**0.93**	–	0.15
Stress	**0.80**	−0.21	–
N Alcohol	–	−0.11	–
Sleep	–	–	–
Walking	–	0.18	0.22
Physical Activity	–	0.22	–
